# Next Frontier in HER2+/HR+ Breast Cancer: Leveraging Cell Cycle Control with CDK4/6 Inhibitors

**DOI:** 10.3390/jpm16030143

**Published:** 2026-03-03

**Authors:** Ilaria Poli, Gaia Rachele Oliva, Ginevra Mongelli, Angelachiara Rotondi, Valentina Frescura, Giorgia Arcuri, Giovanna Garufi, Letizia Pontolillo, Luca Mastrantoni, Elena Di Monte, Noemi Maliziola, Maria Antonia Fucile, Francesca Salvatori, Rita Mondello, Antonella Palazzo, Alessandra Fabi, Emilio Bria, Giampaolo Tortora, Armando Orlandi

**Affiliations:** 1 Unit of Oncology, Comprehensive Cancer Center, Fondazione Policlinico Universitario Agostino Gemelli IRCCS, 00168 Rome, Italy; ilaria.poli@guest.policlinicogemelli.it (I.P.); gaiarachele.oliva@guest.policlinicogemelli.it (G.R.O.); ginevra.mongelli@guest.policlinicogemelli.it (G.M.); angelachiara.rotondi@guest.policlinicogemelli.it (A.R.); valentina.frescura@guest.policlinicogemelli.it (V.F.); giorgia.arcuri@guest.policlinicogemelli.it (G.A.); giovanna.garufi@guest.policlinicogemelli.it (G.G.); letizia.pontolillo@unicatt.it (L.P.); luca.mastrantoni01@icatt.it (L.M.); elena.dimonte@guest.policlinicogemelli.it (E.D.M.); noemimaliziola9@gmail.com (N.M.); mariaantonia.fucile@guest.policlinicogemelli.it (M.A.F.); f.salvatori@uniroma1.it (F.S.); rita.mondello89@gmail.com (R.M.); antonella.palazzo@policlinicogemelli.it (A.P.); giampaolo.tortora@policlinicogemelli.it (G.T.); 2 Dipartimento di Medicina e Chirurgia Traslazionale, Università Cattolica del Sacro Cuore, 00168 Rome, Italy; emilio.bria@policlinicogemelli.it; 3 Liquid Biopsy Platform, Department of Medicine, Division of Hematology-Oncology, Weill Cornell Medicine, New York, NY 10021, USA; 4 Precision Medicine in Breast Cancer Unit, Department of Woman and Child Health and Public Health, Fondazione Policlinico Universitario Agostino Gemelli IRCCS, 00168 Rome, Italy; alessandra.fabi@policlinicogemelli.it; 5 Medical Oncology Unit, Ospedale Isola Tiberina-Gemelli Isola, 00186 Rome, Italy

**Keywords:** breast neoplasms, HER2-positive, hormone receptors, CDK4/6 inhibitors, cell cycle, targeted therapy, precision oncology

## Abstract

HER2-positive/hormone-receptor-positive breast cancer represents approximately 10% of all breast cancer cases and constitutes a distinct biological entity with unique therapeutic challenges. The complex crosstalk between HER2 and estrogen receptor signaling pathways contributes to both primary and acquired resistance to anti-HER2 therapies, and the convergence of these pathways on cell cycle regulation, particularly through the cyclin D1-CDK4/6-Rb axis, has provided a compelling rationale for combining CDK4/6 inhibitors with anti-HER2 therapy. This scoping review aimed to map preclinical and clinical evidence evaluating combinations of CDK4/6 inhibitors with HER2-targeted therapy in HER2+/HR+ disease. Eligible sources included preclinical models and clinical studies assessing CDK4/6 inhibitor-based combinations with anti-HER2 therapy, identified through searches of PubMed, Embase, Cochrane Library, Web of Science and ClinicalTrials.gov. Data were charted and synthesized descriptively according to PRISMA-ScR guidelines. Preclinical studies have demonstrated synergistic antitumor activity when CDK4/6 inhibitors are combined with trastuzumab, pertuzumab, or newer HER2-targeted agents across multiple HER2+ breast cancer models. In the metastatic setting, phase II trials including MonarcHER and PATRICIA II have shown encouraging efficacy signals, while the phase III PATINA trial demonstrated a clinically meaningful 15.2-month progression-free survival benefit with palbociclib plus anti-HER2 therapy and endocrine therapy. In the neoadjuvant setting, trials including NA-PHER2 and MUKDEN-01 demonstrated marked Ki67 suppression and promising pathologic responses, supporting the exploration of chemotherapy de-escalation strategies. Despite these advances, key challenges remain including the identification of predictive biomarkers, optimal treatment sequencing, and the integration of emerging HER2-targeted agents such as trastuzumab deruxtecan. Novel CDK4/6 inhibitors including dalpiciclib and next-generation agents are expanding therapeutic options, while combination strategies incorporating CDK7 inhibition represent future therapeutic frontiers. The evolving landscape of HER2+/HR+ breast cancer treatment increasingly emphasizes precision medicine approaches that leverage cell cycle control mechanisms to overcome resistance and improve patient outcomes across all disease stages.

## 1. Introduction

Breast cancer remains the most frequently diagnosed malignancy among women worldwide, with over 2.3 million new cases diagnosed annually and representing a leading cause of cancer-related mortality despite significant therapeutic advances over recent decades [1,2]. The molecular classification of breast cancer has revolutionized our understanding of this heterogeneous disease, leading to the development of targeted therapies that have dramatically improved patient outcomes [3]. Among the distinct molecular subtypes, HER2-positive (HER2+) breast cancer accounts for approximately 15–20% of all breast cancer cases and was historically associated with aggressive tumor behavior and poor prognosis before the advent of HER2-directed therapies [3].

The introduction of trastuzumab in 1998 marked a paradigm shift in HER2+ breast cancer treatment, transforming a disease with limited therapeutic options into one with multiple effective targeted therapies [4,5]. Subsequent developments including pertuzumab, trastuzumab emtansine (T-DM1), neratinib, and most recently trastuzumab deruxtecan (T-DXd) have further improved outcomes across all disease stages [6,7]. However, within the HER2+ breast cancer population lies significant biological heterogeneity, particularly regarding hormone receptor (HR) status, which profoundly influences treatment responses and clinical outcomes [8,9].

HER2-positive/hormone-receptor-positive (HER2+/HR+) breast cancer, also referred to as “triple-positive” breast cancer, represents approximately 10% of all breast cancer cases and 50–60% of HER2+ tumors [10,11]. This subtype is characterized by overexpression or amplification of HER2 alongside expression of the estrogen receptor (ER) and/or progesterone receptor (PR), creating a unique molecular profile with distinct therapeutic implications [12]. Unlike HER2-enriched tumors that are primarily driven by HER2 signaling, HER2+/HR+ tumors exhibit significant crosstalk between growth factor and steroid hormone signaling pathways, leading to complex resistance mechanisms that challenge current therapeutic approaches [13,14].

The clinical behavior of HER2+/HR+ breast cancer differs substantially from other subtypes. These tumors typically demonstrate lower pathologic complete response (pCR) rates to neoadjuvant chemotherapy plus anti-HER2 therapy compared to HR-negative/HER2+ tumors, with rates ranging from 20–40% versus 60–70% respectively [15,16,17]. However, patients with HER2+/HR+ tumors often exhibit superior long-term survival outcomes, particularly when optimal endocrine therapy is incorporated into treatment regimens [15,16]. This apparent paradox reflects the complex interplay between proliferative HER2-driven signaling and more indolent HR-positive biology [17].

Traditional treatment approaches for HER2+/HR+ breast cancer have largely followed strategies developed for HR-negative disease, utilizing intensive chemotherapy regimens combined with dual HER2 blockade [18,19]. However, growing evidence suggests that the unique biology of this subtype may benefit from alternative therapeutic strategies that specifically target the convergent signaling pathways driving tumor growth and resistance [20,21]. The recognition that both HER2 and ER signaling converge on cell cycle regulatory mechanisms, particularly the cyclin D1-CDK4/6-retinoblastoma (Rb) pathway, has provided compelling rationale for investigating CDK4/6 inhibitors in this population [22,23].

CDK4/6 inhibitors, including palbociclib, ribociclib, and abemaciclib, have demonstrated remarkable efficacy in HR+/HER2- metastatic breast cancer when combined with endocrine therapy, leading to their incorporation into standard treatment guidelines [24,25,26,27]. These agents work by inhibiting the phosphorylation of Rb protein, thereby preventing cell cycle progression from G1 to S phase and inducing cell cycle arrest [28]. The downstream effects of HER2 activation include upregulation of cyclin D1 and enhanced CDK4/6 activity, suggesting that CDK4/6 inhibition could provide synergistic antitumor effects when combined with HER2-targeted therapy [29,30].

The evolving landscape of HER2+/HR+ breast cancer treatment increasingly emphasizes precision medicine approaches that consider the molecular characteristics unique to this subtype. Recent clinical trials have begun to explore the integration of CDK4/6 inhibitors with anti-HER2 therapy, yielding promising results that challenge traditional treatment paradigms [31,32]. Furthermore, the advent of novel HER2-targeted agents and next-generation CDK inhibitors provides unprecedented opportunities to optimize therapeutic strategies for this challenging patient population [33,34].

This comprehensive review examines the current state of CDK4/6 inhibitor development in HER2+/HR+ breast cancer, analyzing the biological rationale, preclinical evidence, clinical trial data, and future therapeutic directions. We aim to provide clinicians and researchers with a thorough understanding of how cell cycle control mechanisms can be leveraged to improve outcomes for patients with this distinct breast cancer subtype.

While our work does not include a formal critical appraisal, it offers added value by assembling and synthesizing the most current clinical and translational data, thereby giving readers an up-to-date and accessible overview of a rapidly evolving therapeutic area. Compared to existing reviews that predominantly address either HER2-directed therapy or CDK4/6 inhibitors in HR+/HER2- disease separately, this work provides an integrated perspective specifically focused on the HER2+/HR+ subtype. We critically synthesize preclinical mechanistic studies, phase II exploratory trials, and the pivotal phase III PATINA data, offering clinicians a comprehensive framework for understanding how the biological crosstalk between pathways translates into therapeutic opportunities and clinical decision-making.

## 2. Materials and Methods

### 2.1. Search Strategy and Selection Criteria

This scoping review follows the Preferred Reporting Items for Systematic Reviews and Meta-Analyses extension for Scoping Reviews (PRISMA-ScR) guidelines (see Supplementary Materials). Although a protocol for this scoping review was developed a priori to guide the study design, literature search, and data charting processes, it was not formally registered in any publicly accessible database. All methods are described in detail in this section to ensure transparency and reproducibility.

A comprehensive literature search was conducted across multiple electronic databases including PubMed/MEDLINE, Embase, Cochrane Library, Web of Science, and ClinicalTrials.gov from inception to February 2025. Grey literature sources, including conference abstracts from major oncology meetings (ASCO, ESMO, SABCS), were screened manually.

The following search terms were used in various combinations: “HER2-positive breast cancer,” “HR-positive breast cancer,” “HER2+/HR+,” “CDK4/6 inhibitor,” “CDK4/6i,” “palbociclib,” “ribociclib,” “abemaciclib,” “dalpiciclib,” “trastuzumab,” “pertuzumab,” “T-DM1,” “T-DXd,” “neratinib,” “pyrotinib,” “tucatinib,” “resistance mechanisms,” “cell cycle,” “biomarkers,” “clinical trials,” and “early breast cancer” or “metastatic breast cancer.”

Studies were eligible if they met the following criteria: (1) preclinical or clinical studies investigating CDK4/6 inhibitors in HER2+/HR+ breast cancer; (2) published in English language; (3) original research or review articles in peer-reviewed journals, abstracts from major oncology conferences, or registered clinical trials with preliminary/final results. Studies focusing exclusively on HER2-negative breast cancer or investigating CDK4/6 inhibitors without anti-HER2 therapy were excluded.

**Figure 1:**
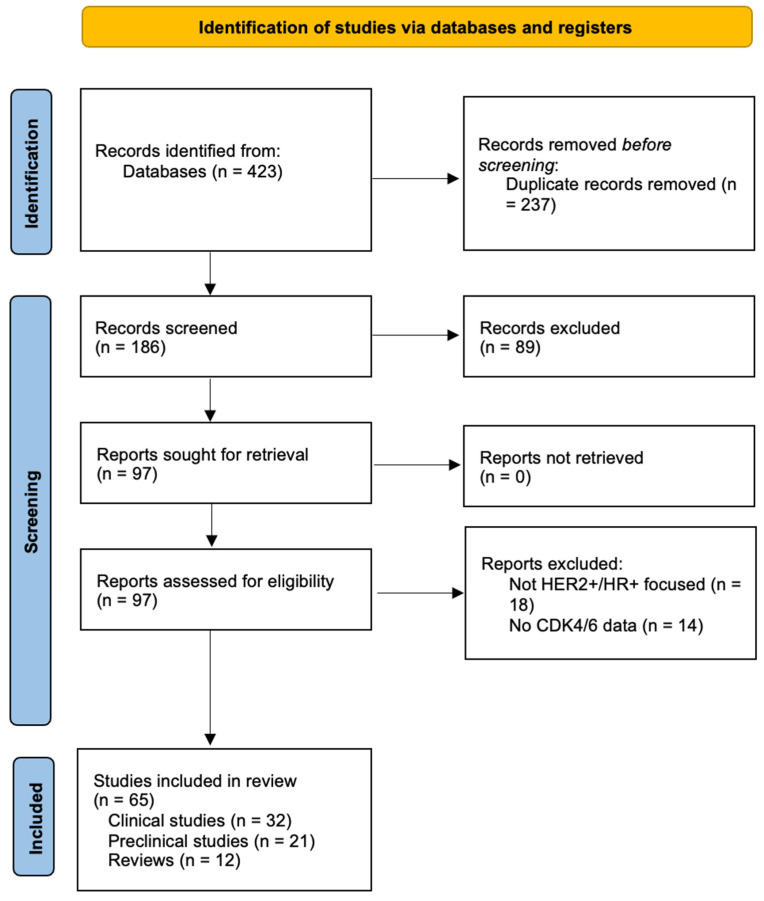
PRISMA Flow Diagram.

Two independent reviewers screened titles, abstracts, and full texts for eligibility, with discrepancies resolved by consensus or by a third reviewer. The search yielded 423 initial records, with 186 remaining after duplicate removal. After title and abstract screening, 97 full-text articles were assessed, resulting in 65 studies included in the final review. The final 65 studies were ultimately included in the qualitative synthesis, consisting of: 32 clinical studies, 21 preclinical studies, 12 review articles. PRISMA flow diagram is reported in Figure 1. Of the 32 trials included in this review, 12 investigated CDK4/6 inhibitors in metastatic HER2+/HR+ disease, directly addressing our objective of mapping current clinical efficacy and safety data. Seven trials focused on neoadjuvant therapy, providing insight into potential chemotherapy de-escalation strategies.

### 2.2. Data Extraction and Analysis

Data extraction was performed using a standardized form capturing: study type (preclinical, clinical); study design; patient population/experimental model; intervention details; comparator (if applicable); outcomes measured; and key findings. For clinical trials, additional information on trial phase, sample size, primary/secondary endpoints, efficacy outcomes, and safety data was extracted.

Study characteristics (e.g., trial design, measures used, population, key findings) are available in Table 1, Table 2 and Table 3.

Quality assessment was conducted using the Quality Assessment Tool for Studies with Diverse Designs (QATSDD) for methodological rigor. No formal meta-analysis was performed due to the heterogeneity of study designs and outcomes.

## 3. Molecular Biology and Mechanistic Rationale

### 3.1. HER2 Signaling Pathway and Cell Cycle Integration

The human epidermal growth factor receptor 2 (HER2), encoded by the ERBB2 gene located on chromosome 17q21, belongs to the HER family of receptor tyrosine kinases that includes HER1 (EGFR), HER3, and HER4 [29]. HER2 is a 185 kDa transmembrane glycoprotein consisting of an extracellular ligand-binding domain, a single transmembrane region, and an intracellular tyrosine kinase domain [33,35]. Unlike other HER family members, HER2 lacks direct ligand-binding capability but serves as the preferred heterodimerization partner, particularly with HER3, forming highly active signaling complexes [33].

**Figure 2:**
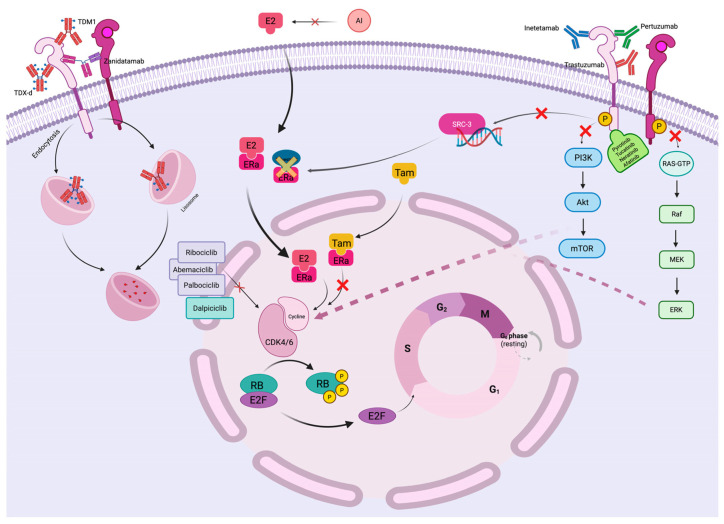
Molecular mechanisms of HER2 and hormone receptor signaling convergence on cell cycle regulation. The diagram illustrates the complex interplay between HER2-activated pathways (PI3K/AKT and MAPK) and estrogen receptor signaling, both of which converge on cyclin D1-CDK4/6-Rb pathway regulation. CDK4/6 inhibitors block this convergent signaling, preventing Rb phosphorylation and cell cycle progression from G1 to S phase. Created in https://BioRender.com (accessed on 22 August 2025).

HER2 activation triggers multiple downstream signaling cascades, most notably the phosphatidylinositol 3-kinase (PI3K)/AKT/mTOR pathway and the mitogen-activated protein kinase (MAPK) pathway (Figure 2) [33]. These pathways converge on cell cycle regulatory mechanisms through multiple mechanisms. The PI3K/AKT pathway promotes cyclin D1 transcription and protein stability while simultaneously inhibiting cell cycle checkpoint proteins including p21 and p27 [36]. The MAPK pathway enhances cyclin D1 gene expression through activation of transcription factors including c-Myc and AP-1 [36].

In HER2-amplified tumors, sustained HER2 signaling leads to constitutive activation of these pathways, resulting in accelerated cell cycle progression and enhanced proliferation [33]. Cyclin D1, a key cell cycle regulator, is frequently overexpressed in HER2+ breast cancer and serves as a critical node integrating multiple growth signals [33].

The formation of cyclin D1-CDK4/6 complexes leads to phosphorylation of the retinoblastoma (Rb) protein, relieving its inhibitory effects on E2F transcription factors and allowing S phase entry (Figure 2) [37].

### 3.2. Hormone Receptor Signaling and Cell Cycle Control

Estrogen receptor signaling profoundly influences cell cycle regulation through both genomic and non-genomic mechanisms [38]. Upon estrogen binding, ER undergoes conformational changes that facilitate DNA binding and transcriptional activation of target genes including cyclin D1, cyclin E1, and c-Myc (Figure 2) [38,39]. Additionally, ER can activate growth factor signaling pathways through membrane-associated ER complexes, creating bidirectional crosstalk with HER2 signaling [40,41].

The convergence of ER and HER2 signaling on cyclin D1 expression creates a particularly potent proliferative stimulus in HER2+/HR+ tumors. Both pathways enhance cyclin D1 transcription through distinct but overlapping mechanisms, leading to amplified CDK4/6 activity and accelerated cell cycle progression (Figure 2) [21,22]. This convergent signaling provides strong biological rationale for targeting CDK4/6 in the HER2+/HR+ population.

### 3.3. CDK4/6-Rb Pathway Regulation

The cyclin D1-CDK4/6-Rb pathway represents a critical cell cycle checkpoint that integrates multiple growth and antiproliferative signals [42]. Under normal conditions, CDK4/6 activity is tightly regulated by several mechanisms including cyclin partner availability, subcellular localization, and inhibitory proteins such as p16, p21, and p27 [43]. In cancer cells, these regulatory mechanisms are frequently disrupted, leading to uncontrolled cell cycle progression [43].

CDK4/6 inhibitors including palbociclib, ribociclib, and abemaciclib are ATP-competitive small molecules that selectively bind to the ATP-binding pocket of CDK4 and CDK6 [44]. By preventing CDK4/6 kinase activity, these agents block Rb phosphorylation, maintaining Rb in its hypophosphorylated state where it sequesters E2F transcription factors (Figure 2) [44]. This results in G1/S cell cycle arrest and reduced proliferation without immediately inducing apoptosis.

### 3.4. Resistance Mechanisms and Therapeutic Implications

Resistance to anti-HER2 therapy in HER2+/HR+ breast cancer involves multiple mechanisms that often intersect with cell cycle regulation [15]. Primary resistance mechanisms include HER2 mutations, splice variants (such as p95HER2), and activation of alternative receptor tyrosine kinases including HER3 and insulin-like growth factor 1 receptor [15]. Secondary resistance develops through acquired mutations, pathway reactivation, or cellular plasticity mechanisms [45].

A key resistance mechanism involves upregulation of cyclin D1 and enhanced CDK4/6 activity, which can overcome the growth inhibitory effects of HER2 blockade [20]. Studies have demonstrated that HER2-targeted therapy-resistant cells frequently exhibit elevated cyclin D1 levels and remain dependent on CDK4/6 activity for proliferation [20]. This dependency provides therapeutic vulnerability that can be exploited through CDK4/6 inhibition.

The presence of hormone receptors adds additional complexity to resistance mechanisms. Estrogen receptor signaling can provide alternative proliferative signals that bypass HER2 blockade [41]. However, the convergence of both pathways on CDK4/6 suggests that cell cycle inhibition could provide broader therapeutic benefit than targeting either pathway alone [41].

## 4. Preclinical Evidence for Cdk4/6 Inhibitors in HER2+ Breast Cancer

### 4.1. Cell Line and Xenograft Studies

Extensive preclinical investigations have established the therapeutic potential of CDK4/6 inhibitors in HER2+ breast cancer models.

Finn et al. performed comprehensive cell line screening studies demonstrating that palbociclib preferentially inhibited proliferation in luminal ER+ breast cancer cell lines, including those with HER2 overexpression [46]. The study revealed synergistic interactions between palbociclib and trastuzumab in HER2+ cell lines, with combination treatment resulting in enhanced G1 arrest and reduced cell viability compared to either agent alone [46] (Table 1).

**Table 1:** Summarizing preclinical models and in vitro/in vivo results.

Model	Key Findings	Mechanism & Additional Insights	Reference
MMTV-neu (MMTV-ErbB2/HER2) transgenic mouse; human HER2+ breast cancer cell lines HCC1954 and MDA-MB-453	Resistance to HER2 therapy linked to nuclear cyclin D1 and CDK4; CDK4/6 inhibition restored sensitivity and had a synergistic antitumor effect.	Abemaciclib restored EGFR kinase family signaling by activating Akt kinase and TSC2 (mTOR inhibitor), reducing mTOR-mediated feedback inhibition.	Goel S. et al. (2016) [20]
Panel of 47 human breast cancer cell lines, including luminal ER+ (MCF-7, T-47D, ZR-75-1, BT-474) and HER2-amplified (SK-BR-3, MDA-MB-361, UACC-893) models; compared with basal-like and triple-negative lines (MDA-MB-231, BT-549, HCC-38, Hs578T)	Palbociclib selectively inhibited luminal ER+ breast cancer cells, including HER2+ subtypes; non-luminal/basal subtypes were resistant.	Synergistic effect with tamoxifen (ER+ lines) and trastuzumab (HER2+ lines). Induced G1 cell cycle arrest by inhibiting RB phosphorylation.	Finn RS. et al. (2009) [46]
Panel of 44 cell lines including Luminal ER+ HER2+ (including ZR-75-30, UACC-732, HCC1419, UACC-812, BT-474) and TN breast cancer cell lines (including MDA-MB-231, BT-549, HCC-38, Hs578T) and xenograft models	Abemaciclib showed significant activity in both luminal ER+ and HER2-amplified cancer cells, especially those with high ER and intact Rb signaling. Specific subgroups of TNBC cell lines matching the biomarker profile of intact Rb signaling were also sensitive.	Abemaciclib combined effectively with trastuzumab and in triple combination with tamoxifen induced significant regressions in both trastuzumab-sensitive and -resistant HER2+/ER+ xenografts. Abemaciclib maintained activity in HER2-amplified tumors progressing on trastuzumab.	O’Brien N. et al. (2018) [47]
Human HER2-amplified breast cancer cell lines (SK-BR-3, BT-474, HCC1954, MDA-MB-361) and matched controls	HER2-driven signaling regulates E2F1-dependent DNA metabolism and gene replication.	HER2 phosphorylates SRC-3, a transcriptional coactivator, to enhance E2F1 activity, promoting proliferation. Palbociclib and lapatinib inhibited E2F1 and DNA synthesis.	Nikolai BC. et al. (2016) [29]
Ex vivo cultured primary human breast tumor tissues; validation performed in breast cancer cell lines (MCF-7, T-47D, BT-474, SK-BR-3, MDA-MB-231)	CDK4/6 inhibition suppressed proliferation in ~85% of cases, regardless of ER or HER2 status.	Tumor samples showed variable sensitivity, suggesting a potential biomarker-driven selection for CDK4/6i therapy.	Dean JL. et al. (2012) [48]
HER2-amplified human breast cancer cell lines (BT-474, SK-BR-3, HCC1954, MDA-MB-361) and nude-mouse xenograft models derived from BT-474 cells	Palbociclib + pyrotinib showed synergistic anti-tumor activity.	Combination reduced pAKT and pHER3 activation, leading to G0-G1 cell cycle arrest and increased apoptosis, with no significant toxicity increase.	Zhang K. et al. (2019) [30]
Human HER2-amplified breast cancer cell lines (BT-474, SK-BR-3, and MCF10A engineered to express HER2) + primary breast tumor explants + HER2-positive xenograft models	CDK4/6i enhanced TKIs (neratinib, afatinib) in an additive manner.	CDK4/6i suppressed tumor growth more effectively when combined with HER2 TKIs.	Witkiewicz AK. et al. (2014) [49]
HER2-amplified human breast cancer cell lines (BT-474, SK-BR-3, HCC1954) and HER2+ patient-derived xenograft (PDX) models	Neratinib and palbociclib combination significantly improved tumor volume reduction.	Targeted multiple pathways, including cell cycle inhibition and HER2 signaling suppression.	Zhao M. et al. (2021) [50]
HER2+ breast cancer cell lines (BT-474, SKBr3, MDA-MB-361)	Palbociclib has an inhibitory effect in resistant and non-resistant HER2-positive cell lines suggesting palbociclib represents an alternate targeting pathway for growth inhibition.	No reliable biomarker to predict palbociclib effects was identified.	ElChaarani B. et al. (2017) [51]
HER2-E tumor samples and HER2+ breast cancer cell lines (BT474, SKBR3, HCC1954, BT474-L^R^T^R^, BT474-Tu^R^T^R^)	HER2-E tumors cells that are sensitive to anti-HER2 therapy but do not die acquire a Luminal A phenotype leading to anti-HER2 resistance but become more sensitive to palbociclib.	Subtype switching from HER2-E to Luminal A seems reversible and might open an opportunity to treat the acquired phenotype with drugs such as endocrine therapy and/or CDK4/6 inhibitors.	Brasó-Maristany F. et al. (2020) [52]
HER2+ and HER2- tumor samples	HER2 positivity leads to significantly higher levels of CDK4/6 activity.	Among HER2+ cases, there was a trend of positive correlation between the HER2 gene copy number and the pRb level, although not statistically significant.	Sinclair W.D. et al. (2022) [53]

O’Brien et al. conducted a comprehensive analysis of the preclinical activity of abemaciclib, alone or in combination, in a panel of 44 breast cancer cell lines [47]. Results showed HER2-amplified cell lines to be highly responsive to abemaciclib, especially those with higher levels of ER protein accompanied by intact downstream Rb sigaling (Table 1). Nonetheless, strong correlations were identified between response to abemaciclib and two other main CDK4/6 inhibitors, palbociclib and ribociclib.

Goel and colleagues conducted seminal studies using transgenic mouse models of HER2+ breast cancer, demonstrating that tumors resistant to HER2-targeted therapy exhibited elevated nuclear cyclin D1 and CDK4 expression [20]. Importantly, these resistant tumors remained sensitive to CDK4/6 inhibition, and combination therapy with abemaciclib enhanced the antitumor efficacy of HER2-targeted agents [20] (Table 1).

Zhang and colleagues investigated the combination of palbociclib with pyrotinib, an irreversible pan-HER tyrosine kinase inhibitor, in HER2+ breast cancer models [30]. The combination demonstrated synergistic antitumor activity with significant reductions in phosphorylated AKT and HER3 levels, leading to G0/G1 cell cycle arrest and increased apoptosis without notable toxicity enhancement [30] (Table 1).

Patient-derived xenograft (PDX) models have provided particularly valuable insights into the clinical translatability of CDK4/6 inhibitor combinations. Zhao et al. demonstrated that the combination of neratinib and palbociclib significantly improved tumor volume reduction in HER2+ PDX models compared to either agent alone [50]. The synergistic effects were associated with enhanced suppression of both cell cycle progression and HER2 signaling pathways [49] (Table 1).

### 4.2. Molecular Mechanism Studies

Detailed mechanistic studies have elucidated the molecular basis for synergy between CDK4/6 inhibitors and anti-HER2 therapy. Nikolai and colleagues demonstrated that HER2 signaling regulates E2F1-dependent DNA metabolism and gene replication through phosphorylation of steroid receptor coactivator-3 (SRC-3) (Figure 2) [29]. Both lapatinib and palbociclib disrupted E2F1 activity and its target genes, significantly limiting de novo DNA synthesis and cell proliferation [29] (Table 1).

El-Chaarani et al. conducted comprehensive studies examining CDK4/6 inhibitor activity in both trastuzumab-sensitive and resistant HER2+ cell lines. Palbociclib demonstrated inhibitory effects across all tested models, suggesting that CDK4/6 dependence persists despite HER2 therapy resistance [51,54] (Table 1). These findings support the concept that CDK4/6 inhibition can overcome resistance mechanisms that develop during anti-HER2 treatment.

Studies by Witkiewicz et al. revealed complementary mechanisms of action when CDK4/6 inhibitors are combined with HER2 tyrosine kinase inhibitors. The investigators demonstrated additive antiproliferative effects across various HER2+ models, with enhanced tumor growth suppression when CDK4/6 inhibition was combined with neratinib or afatinib [49] (Table 1).

### 4.3. Biomarker Discovery Studies

Preclinical investigations have identified potential predictive biomarkers for CDK4/6 inhibitor efficacy in HER2+ breast cancer. Dean et al. utilized ex vivo breast tumor tissue models to examine cytostatic responses to palbociclib, demonstrating that CDK4/6 inhibition effectively suppressed cellular proliferation in approximately 85% of cases regardless of ER or HER2 status [48]. However, the degree of growth inhibition varied significantly between samples, suggesting the potential for biomarker-driven patient selection [48] (Table 1).

Sinclair and colleagues investigated the relationship between HER2 gene copy number and CDK4/6 activity in HER2+ breast cancer. Their analysis revealed a correlation between higher HER2 gene amplification and increased CDK4/6 dependency, suggesting that HER2 copy number might serve as a predictive biomarker for CDK4/6 inhibitor efficacy [53,55] (Table 1).

Studies examining molecular subtypes within HER2+ disease have revealed differential CDK4/6 inhibitor sensitivity. Brasó-Maristany et al. demonstrated that HER2-enriched tumors undergo phenotypic switching to luminal-like characteristics during anti-HER2 treatment, potentially increasing susceptibility to CDK4/6 inhibition (Table 1). This plasticity suggests that combination strategies might be particularly effective in preventing or exploiting subtype switching [52].

## 5. Clinical Evidence in Advanced and Metastatic Disease

### 5.1. Phase II Clinical Trials

#### 5.1.1. MonarcHER Trial

**Figure 3:**
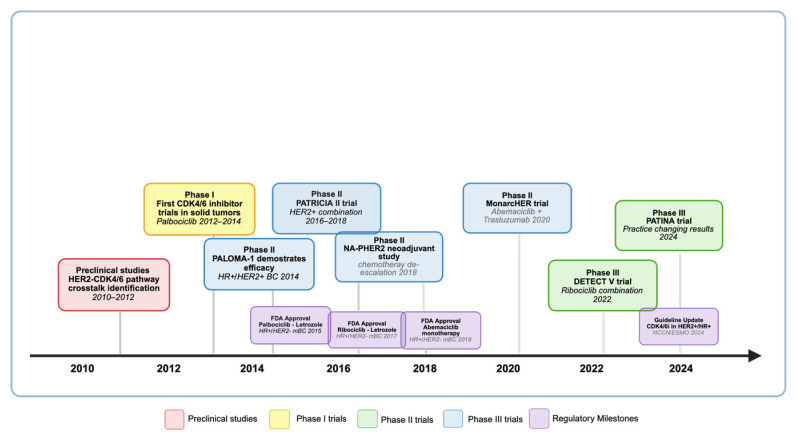
Clinical development timeline of CDK4/6 inhibitors in HER2+/HR+ breast cancer. This comprehensive timeline shows the evolution of clinical trials from early preclinical studies through current phase III trials, highlighting key milestones and regulatory approvals. Created in https://BioRender.com. (accessed on 20 August 2025).

The MonarcHER study represents the most comprehensive phase II evaluation of CDK4/6 inhibitors in HER2+/HR+ metastatic breast cancer (Table 2). This randomized trial enrolled 237 patients across three treatment arms: abemaciclib plus trastuzumab and fulvestrant (arm A), abemaciclib plus trastuzumab (arm B), and standard-of-care chemotherapy plus trastuzumab (arm C) (Figure 3) [31].

The primary-endpoint analysis demonstrated that arm A achieved a median progression-free survival (PFS) of 8.3 months compared to 5.7 months in arm C (hazard ratio [HR] 0.673, 95% CI 0.45–1.00), representing a clinically meaningful improvement [31]. Overall survival analysis showed favorable trends in the abemaciclib-containing arms, with median overall survival not reached in arm A versus 22.9 months in arm C. Biomarker analyses revealed that patients with luminal A or B PAM50 subtypes derived greater benefit from CDK4/6 inhibitor therapy [31].

Safety analysis demonstrated manageable toxicity profiles with expected class effects of CDK4/6 inhibitors including neutropenia, diarrhea, and fatigue. Importantly, the combination did not result in significant increases in cardiac toxicity or other HER2-targeted therapy-related adverse events [31].

**Table 2:** Summary of clinical trials investigating the role of CDK4/6i in HR+/HER2+ metastatic breast cancer.

Trial	Phase	*n*	Treatments	Primary-Endpoint Results	Reference
**NCT01976169**	I/Ib	29	Palbociclib + T-DM1	MTD: NR ORR 33% (95% CI: 13–59%)	-
**LORDSHIPS (NCT03772353)**	Ib	59	Dalpiciclib + pyrotinib + ET	ongoing	Zhang, J. et al. (2022) [56]
**NCT02657343**	Ib/II	25	Cohort A: ribociclib + T-DM1 (3+3 dose escalation-design) Cohort B: ribociclib + H Cohort C: ribociclib + H + Fulvestrant	CBR: NR	-
**NCT03913234**	Ib/II	95	Letrozole + H + ribociclib	ongoing	-
**NCT04224272**	IIa	51	Zanidatamab + palbociclib + fulvestrant	PFS6 *n* (%; 95% CI): 34 (66.7%; 52.1–79.2) Median progression-free survival (95% CI), months: 11.7 (8.4–14.9) Confirmed ORR (in 46 patients with measurable disease): 16 (34.8%; 21.4–50.2)	-
**PATRICIA II (NCT02448420)**	II	73	Cohort A (HR-): palbociclib + H Cohort B1 (HR+): palbociclib + H Cohort B2 (HR+): palbociclib + H + ET Cohort C1: palbociclib + H + ET Cohort C2: treatment based on physician’s choice	PFS6 (cohorts A–B1–B2) A: 33.3%, 10.8–77.8 B1: 42.9%, 24.5–62.86 B2: 46.4%, 27.5–66.1 Investigator-assessed median PFS (cohorts C1–C2) (HR = 0.52, 95% CI: 0.29–0.95) C1: 9.1 months (5.9–17.6) C2: 7.5 months (5.5–11.1)	Ciruelos, E. et al. (2020) [57]
**MonarcHER (NCT02675231)**	II	237	Arm A: abemaciclib + H + fulvestrant Arm B: abemaciclib + H Arm C: CT + H	PFS between A and C 8.3 vs. 5.7 months (HR = 0.673, 95% CI: 0.45–1.00) PFS between B and C 5.7 vs. 5.7 months (HR = 0.94, 95% CI: 0.64–1.38)	Tolaney S.M. et al. (2020) [58]
**DAP-HER-01 (NCT04293276)**	II	41	Dalpiciclib + pyrotinib	Overall ORR 70% Median PFS 11 months (95% CI: 7.3–19.3) Median PFS (patients with brain metastases) 11 months (95% CI: 5.4-NA)	Yan M. et al. (2023) [34]
**DAP-HER-02 (NCT05328440)**	II	120	Arm A: pyrotinib + dalpiciclib + fulvestrant Arm B: pyrotinib + dalpiciclib + inetetamab	ongoing	Wang X. et al. (2024) [59]
**NCT04095390**	II	60	Arm A: pyrotinib + dalpiciclib + letrozole Arm B: pyrotinib + dalpiciclib + capecitabine Arm C: pyrotinib + dalpiciclib	ongoing	-
**PATINA (AFT-38/NCT02947685)**	III	518	Arm A: palbociclib + H ± P + ET Arm B: H ± P + ET	Median PFS increased from 29.1 to 44.3 months (Δ 15.2 months) (HR = 0.74, 95% CI: 0.58–0.94)	Loibl, S. et al. (2018) [32]
**DETECT V (NCT02344472)**	III	271	CT + H + P + ribociclib ET + H + P + ribociclib	Addiction of ribociclib to CT or ET: Median OS not reached vs. 46.1 months (HR = 0.42, 95% CI: 0.24–0.74) Median PFS 27.2 vs. 15.6 months (HR = 0.52, 95% CI: 0.37–0.75)	Janni, W. et al. (2024) [60]

PFS (progression-free survival), HR (hazard ratio), CI (confidence interval), PFS6 (progression-free survival rate at 6 months), H (trastuzumab), P (pertuzumab), CT (chemotherapy), ET (endocrine therapy), HR- (hormone-receptor negative), HR+ (hormone-receptor-positive), CBR (clinical benefit rate), NR (not reached), OS (overall survival), NA (not assessed), MTD (maximum tolerated dose), ORR (objective overall response rate), Δ (PFS gain)

#### 5.1.2. PATRICIA II Trial

The SOLTI-1303 PATRICIA II study evaluated palbociclib in combination with trastuzumab with or without endocrine therapy in heavily pretreated patients with HER2+ advanced breast cancer (Table 2) (Figure 3) [57]. The trial included multiple cohorts based on hormone receptor status, allowing detailed analysis of the HER2+/HR+ population.

In cohort B2 (HR+ patients receiving palbociclib, trastuzumab, and endocrine therapy), the 6-month progression-free survival rate was 46.4% (95% CI 27.5–66.1), comparing favorably to historical controls [57]. Median PFS reached 9.1 months in the combination cohort versus 7.5 months in patients receiving physician’s choice therapy [57]. PAM50 subtyping revealed particular benefit in luminal A and B subtypes, consistent with the biological rationale for CDK4/6 inhibition in HR+ disease [57].

The study provided important insights into the activity of CDK4/6 inhibitors in trastuzumab-pretreated patients, demonstrating that prior anti-HER2 therapy does not preclude benefit from cell cycle inhibition. This finding supports the concept that CDK4/6 dependence persists despite the development of HER2 therapy resistance [61].

### 5.2. Phase III Clinical Trials

#### 5.2.1. PATINA Trial

The AFT-38/PATINA trial represents a pivotal phase III study that has provided practice-changing evidence for CDK4/6 inhibitors in HER2+/HR+ metastatic breast cancer (Table 2) (Figure 3) [32]. This randomized, open-label trial enrolled 518 patients with HER2+/HR+ advanced breast cancer who had no evidence of disease progression after completing induction chemotherapy.

Patients were randomized 1:1 to receive palbociclib plus anti-HER2 therapy (trastuzumab with or without pertuzumab) plus endocrine therapy versus anti-HER2 therapy plus endocrine therapy alone. The primary endpoint was investigator-assessed progression-free survival.

At the planned interim analysis with a data cutoff in October 2024, the trial met its primary endpoint with a clinically meaningful 15.2-month improvement in median PFS. The combination arm achieved a median PFS of 44.3 months compared to 29.1 months in the control arm (HR 0.74, 95% CI 0.58–0.94, *p* = 0.0214). This represents one of the largest PFS benefits observed in HER2+ breast cancer clinical trials and establishes CDK4/6 inhibition as a new standard-of-care option.

Safety analysis confirmed the expected toxicity profile of palbociclib with no unexpected safety signals when combined with anti-HER2 therapy and endocrine therapy. The most common grade 3/4 adverse events included neutropenia (61% vs. 6%), leukopenia (26% vs. 2%), and anemia (8% vs. 1%) in the combination versus control arms [32].

Biomarker analyses from PATINA are ongoing to provide crucial insights into predictive factors for CDK4/6 inhibitor benefit in the HER2+/HR+ population. Planned correlative studies include PAM50 subtyping, assessment of cell cycle gene expression signatures, and evaluation of ctDNA markers [32].

#### 5.2.2. DETECT V Trial

The randomized phase III DETECT V trial provided additional evidence supporting CDK4/6 inhibitor integration in HER2+/HR+ advanced breast cancer. This study enrolled 271 patients with HER2+/HR+ advanced breast cancer who were randomized to anti-HER2 therapy combined with either physician’s choice endocrine therapy or chemotherapy (Table 2) (Figure 3) [60].

Following the enrollment of 124 patients, the study was amended to add ribociclib to endocrine therapy in both treatment arms, creating an opportunity to assess the impact of CDK4/6 inhibition. The second interim efficacy analysis with a data cutoff in April 2024 demonstrated that ribociclib addition further improved survival outcomes [60].

Patients receiving ribociclib-containing regimens achieved superior overall survival with a median not reached versus 46.1 months in the control group (HR 0.42, 95% CI 0.24–0.74) [60]. Median PFS was also improved at 27.2 months versus 15.6 months (HR 0.52, 95% CI 0.37–0.75) [60]. These results provide additional validation for CDK4/6 inhibitor integration across different treatment contexts.

### 5.3. Novel Combinations and Emerging Agents

#### 5.3.1. Antibody–Drug Conjugate Combinations

The integration of CDK4/6 inhibitors with antibody–drug conjugates (ADCs) represents an emerging therapeutic frontier with significant potential [62]. A phase I/Ib trial investigating the combination of T-DM1 and palbociclib in previously treated HER2+ relapsed patients demonstrated encouraging response rates, confirming that CDK4/6 inhibition can resensitize HER2-resistant breast cancer [62].

Despite its ever-growing importance in breast cancer treatment, there appear to be no current studies on the combination of T-DXd and CDK4/6i in either the metastatic or the early setting. In the case any trial is planned, careful attention to safety will be essential, as the unique toxicity profile of T-DXd, particularly interstitial lung disease, may influence combination feasibility.

#### 5.3.2. Zanidatamab Combinations

Zanidatamab, a bispecific antibody targeting two distinct HER2 epitopes, has shown promising activity in heavily pretreated HER2+ breast cancer. A phase II trial evaluating zanidatamab plus palbociclib and fulvestrant in pretreated HER2+/HR+ patients demonstrated encouraging efficacy signals [63].

Among 51 heavily pretreated patients (50 of whom had prior ADC exposure), the combination achieved a 6-month PFS rate of 66.7% with a median PFS of 11.7 months [63]. The confirmed objective response rate was 34.8% with three complete responses and 13 partial responses [63]. Importantly, 33% of patients remained on treatment for longer than 12 months, suggesting durable disease control [63].

## 6. Neoadjuvant Setting and De-Escalation Strategies

### 6.1. Rationale for Chemotherapy De-Escalation

The neoadjuvant treatment of HER2+/HR+ breast cancer presents unique challenges and opportunities that differ substantially from other breast cancer subtypes [13]. Traditional approaches utilizing intensive chemotherapy regimens combined with dual HER2 blockade yield pathologic complete response (pCR) rates of approximately 20–40% in the HER2+/HR+ population, significantly lower than the 60–70% rates observed in HR-negative disease [64,65,,66].

However, the prognostic significance of pCR in HER2+/HR+ breast cancer remains controversial, with several studies suggesting that the absence of pCR does not necessarily translate to inferior long-term outcomes [67,68]. This observation, combined with the excellent prognosis of many HER2+/HR+ tumors, has led to increased interest in de-escalation strategies that maintain efficacy while reducing treatment-related toxicity.

Notably, several trials attempted chemotherapy descalation in the neoadjuvant setting, reporting considerably low pCR rates [69,70]. Nonetheless, long-term outcomes might not be as extensively impacted. For example, the three-year follow-up of PHERGain showed that the invasive disease-free survival rate was highest in patients who achieved pCR with ET and without chemotherapy. This indicates that pCR might not be the optimal endpoint for evaluating responses to neoadjuvant ET, and future trials should explore alternative indicators such as preoperative endocrine prognostic index and endocrine sensitive disease rate for their clinical significance.

The biological rationale for CDK4/6 inhibitor integration in the neoadjuvant setting stems from the recognition that HER2+/HR+ tumors exhibit lower proliferative indices compared to HER2-enriched tumors [71]. These tumors may be less dependent on intensive cytotoxic therapy and more responsive to targeted approaches that address the specific molecular drivers of growth [72].

### 6.2. NA-PHER2 Trial

The NA-PHER2 study represents the most comprehensive investigation of CDK4/6 inhibitors in neoadjuvant HER2+/HR+ breast cancer to date (Table 3). This exploratory phase II trial evaluated neoadjuvant trastuzumab and pertuzumab plus palbociclib with or without fulvestrant in patients with HER2+ early breast cancer (Figure 3) [65].

**Table 3:** Summary of clinical trials investigating the role of CDK4/6i in HR+/HER2+ early breast cancer.

Study	Phase	CDK 4/6 Inhibitor	HER2 Target Therapy	Endocrine Therapy	Primary Endpoints	Key Findings	Reference
**NA-PHER2**	II	Palbociclib	Trastuzumab and Pertuzumab	Fulvestrant	Ki67 reduction at 2 weeks and at surgery	Reduction in Ki67 from 31.9% at baseline to 4.3% at 2 weeks and 12.1% at surgery. Three patients reached pCR at surgery.	Gianni L. et al. (2018) [65]
**PALTAN**	II	Palbociclib	Trastuzumab	Letrozole	pCR	RCB-0 and RCB-I was achieved in 46.2% of patients.	Ademuyiwa F.O. et al. (2023) [73]
**MUKDEN-01**	II	Dalpiciclib	Pyrotinib	Letrozole	pCR	RCB-0 and RCB-1 in 55.7% of patients	Niu, N. et al. (2022) [74]
**TOUCH**	II	Palbociclib	Trastuzumab and Pertuzumab	Letrozole	pCR	Ongoing	Malorni L. et al. (2026) [75]
**UCLA B-13 (NCT05319873)**	Ib/II	Ribociclib	Trastuzumab and Tucatinib	Fulvestrant	MTD (phase I), pCR (phase II)	Ongoing	/
**NCT05800756**	II	Dalpiciclib	Pyrotinib and Trastuzumab	Letrozole	pCR	Ongoing	/
**MUKDEN 01 Plus (NCT05228951)**	II	Dalpiciclib	Pyrotinib and Trastuzumab	Letrozole	tpCR	pCR rate of 58%, with 75% of patients obtaining RCB-0 and RCB-I.	Huo S. et al. (2024) [76]

The primary endpoint focused on Ki67 reduction at 2 weeks and at surgery, serving as a pharmacodynamic marker of cell cycle inhibition. Among 36 enrolled patients eligible for endpoint analysis, the combination demonstrated remarkable antiproliferative activity with Ki67 levels decreasing from 31.9% at baseline to 4.3% at 2 weeks and 12.1% at surgery [65].

Clinical response rates were impressive, with 97% of patients achieving an objective response, 50% demonstrating complete clinical responses, and 27% attaining pCR at surgery. Notably, 5 patients achieved pCR as early as 2 weeks after treatment initiation, with 3 maintaining this response at final surgery [65].

The study included additional cohorts examining different combinations. Cohort B evaluated the combination without fulvestrant, recognizing the relative resistance of HER2-enriched tumors to endocrine therapy. This cohort achieved similar Ki67 suppression (from 33.4% to 5.5% at 2 weeks) with an 88.5% objective response rate and 19.2% pCR rate [65].

Cohort C specifically evaluated patients with HER2-low tumors, demonstrating the broad applicability of CDK4/6 inhibition across the HER2 expression spectrum. This cohort achieved particularly impressive results with over 90% of tumors showing Ki67 reduction below 10% at 2 weeks and a 78.3% clinical response rate [65].

### 6.3. PALTAN Trial

The PALTAN study provided additional evidence for neoadjuvant CDK4/6 inhibitor efficacy in HER2+/HR+ breast cancer (Table 3). This phase II trial evaluated palbociclib plus letrozole and trastuzumab in patients with clinical stage II-III HER2+/HR+ breast cancer [73].

The primary endpoint was pCR rate, defined as residual cancer burden (RCB) 0. While only 7.7% of patients achieved true pCR, the broader endpoint of RCB 0-I was achieved in 46.2% of patients, suggesting meaningful pathologic responses. The study confirmed significant antiproliferative activity with median Ki67 values decreasing from 27.1% at baseline to 0.38% at cycle 1 day 15 and 4.9% at surgery [73].

Complete cell cycle arrest (defined as Ki67 ≤ 2.7%) was observed in 84.6% of patients at day 15 following palbociclib administration, compared to only 27% at surgery after treatment discontinuation [73]. This pattern suggests that continuous CDK4/6 inhibition may be necessary to maintain optimal cell cycle control.

### 6.4. MUKDEN Trials

The MUKDEN series of trials, conducted primarily in Chinese populations, has provided valuable insights into CDK4/6 inhibitor combinations with novel anti-HER2 agents [77]. MUKDEN-01 evaluated neoadjuvant pyrotinib, letrozole, and dalpiciclib in HER2+/HR+ breast cancer patients [74] (Table 3).

Among 79 patients receiving 5 cycles of neoadjuvant treatment, the combination achieved a 30.4% pCR rate with 55.7% of patients reaching RCB 0-I. Significant Ki67 reduction was observed from 40.4% at baseline to 17.9% at surgery (*p* < 0.001). The MUKDEN-01 Plus trial, incorporating trastuzumab into the regimen, achieved even more impressive results with a 58% pCR rate and 75% RCB 0-I rate [76] (Table 3).

These studies are particularly notable for their inclusion of pyrotinib, an irreversible pan-HER tyrosine kinase inhibitor with superior CNS penetration compared to traditional anti-HER2 agents [78]. The high efficacy observed with pyrotinib-containing regimens suggests that more potent HER2 inhibition may enhance the benefits of CDK4/6 inhibitor combinations [30].

## 7. Predictive Biomarkers and Patient Selection

### 7.1. Genomic Biomarkers

The identification of predictive biomarkers for CDK4/6 inhibitor efficacy in HER2+/HR+ breast cancer remains a critical unmet clinical need. Several genomic alterations have emerged as potential candidates based on mechanistic understanding and preliminary clinical observations [79,80].

Retinoblastoma (RB1) status represents the most extensively studied biomarker for CDK4/6 inhibitor response [81]. Since CDK4/6 inhibitors exert their effects through Rb protein phosphorylation, RB1 loss-of-function mutations or deletions would theoretically confer resistance [81]. However, RB1 alterations are relatively infrequent in breast cancer (<5%), limiting their utility as exclusionary biomarkers [82]. Thus, despite being subject to more extensive research, RB1 loss cannot yet be reliably differentiated from newer exploratory biomarkers with regard to their predictive value in HER2+/HR+ breast cancer.

CCNE1 (cyclin E1) amplification has emerged as a potential resistance mechanism in multiple studies [83]. Guarducci et al. demonstrated that the CCNE1/RB1 expression ratio serves as a more effective predictor of palbociclib resistance than either marker alone [83]. Retrospective analyses from the neoadjuvant NeoPalAna trial confirmed that elevated CCNE1/RB1 ratios distinguish between sensitive and resistant patients [84].

PIK3CA mutations, present in approximately 30–40% of HER2+/HR+ breast cancers, represent another important genomic biomarker [85]. Several studies have suggested that PIK3CA mutations correlate with reduced anti-HER2 therapy efficacy [86,87,88,89,90] and may influence CDK4/6 inhibitor sensitivity [91]. The SOLAR-1 trial demonstrated that PIK3CA-mutated tumors benefit from alpelisib addition to fulvestrant, suggesting potential for triple combinations targeting PI3K, CDK4/6, and HER2 [92].

### 7.2. Transcriptomic Signatures

Gene expression profiling has revealed several transcriptomic signatures associated with CDK4/6 inhibitor sensitivity [93]. Cell cycle gene expression signatures, including the CycleX score and proliferation signatures, correlate with treatment response across multiple studies [94]. However, the relationship between these signatures and outcome appears complex, with some studies suggesting that highly proliferative tumors may be more sensitive to CDK4/6 inhibition [95].

PAM50 molecular subtyping has provided particularly valuable insights in the HER2+ population [96]. Multiple trials, including MonarcHER and PATRICIA II, have demonstrated that luminal A and B subtypes derive greater benefit from CDK4/6 inhibitor therapy compared to HER2-enriched subtypes [57,58]. This observation aligns with the biological understanding that luminal subtypes exhibit greater ER signaling and CDK4/6 dependence.

Interestingly, several studies have observed phenotypic switching from HER2-enriched to luminal characteristics during anti-HER2 therapy. Brasó-Maristany et al. demonstrated that this plasticity may increase susceptibility to CDK4/6 inhibition, suggesting that combination strategies could exploit tumor evolution [52].

### 7.3. Protein Biomarkers

Several protein markers have been investigated as potential predictors of CDK4/6 inhibitor efficacy [97]. Cyclin D1 expression, while commonly elevated in HER2+ breast cancer, has not consistently predicted treatment response [97,98]. This may reflect the complex regulation of CDK4/6 activity that extends beyond cyclin D1 availability.

Ki67 proliferation index has emerged as both a pharmacodynamic marker and potential predictor of response [99]. The NA-PHER2 and PALTAN trials demonstrated dramatic Ki67 suppression with CDK4/6 inhibitor therapy, and the degree of suppression may correlate with clinical benefit [65,73]. However, the optimal Ki67 threshold and timing of assessment remain to be established.

p16 expression, as a surrogate for CDK4/6 pathway activation, has shown promise in preclinical studies but requires further clinical validation [98,100]. Similarly, other cell cycle proteins including p21, p27, and phosphorylated Rb have been investigated but lack consistent predictive value [101,102].

### 7.4. Liquid Biopsy Applications

Serial biopsies and ctDNA monitoring may help identify optimal timing for CDK4/6 inhibitor introduction [103].

Circulating tumor DNA (ctDNA) analysis represents an emerging approach for biomarker discovery and monitoring in HER2+/HR+ breast cancer [104]. Several studies have demonstrated the feasibility of detecting CDK4/6 inhibitor resistance mutations in plasma samples [105]. Common resistance alterations identified through ctDNA include RB1 mutations, CCNE1 amplifications, and pathway reactivation signals [105].

The dynamic nature of ctDNA allows for real-time monitoring of treatment response and resistance development. Studies in HR+/HER2- breast cancer have demonstrated that ctDNA clearance during CDK4/6 inhibitor therapy correlates with improved outcomes [106]. Similar approaches are now being investigated in HER2+ disease to guide treatment decisions [107].

Circulating tumor cells (CTCs) represent another liquid biopsy approach with potential applications. CTC enumeration and molecular characterization may provide insights into tumor heterogeneity and resistance mechanisms [108]. However, the clinical utility of CTC-based biomarkers in the CDK4/6 inhibitor setting requires further investigation [109].

## 8. Safety Considerations and Clinical Management

### 8.1. Toxicity Profile of CDK4/6 Inhibitor Combinations

The integration of CDK4/6 inhibitors with anti-HER2 therapy generally results in manageable toxicity profiles with predictable side effects [32,58,65]. The most common adverse events reflect the known class effects of CDK4/6 inhibitors, primarily hematologic toxicities including neutropenia, leukopenia, and thrombocytopenia [32,58,65].

Neutropenia represents the most frequent grade 3/4 toxicity, occurring in 60–80% of patients receiving combination therapy. However, febrile neutropenia rates remain low (<5%), and most episodes are asymptomatic and manageable with dose modifications. The neutropenia is typically non-cumulative and reversible upon treatment interruption [32,58,65].

Non-hematologic toxicities vary among different CDK4/6 inhibitors but generally include fatigue, gastrointestinal effects, and skin reactions. Nausea and vomiting are less common but may be exacerbated when combined with trastuzumab [32,58,65].

Diarrhea is particularly common with abemaciclib-containing regimens, occurring in up to 70% of patients but rarely requiring treatment discontinuation [58,110].

### 8.2. Drug Interactions and Pharmacological Considerations

CDK4/6 inhibitors exhibit distinct pharmacokinetic profiles that influence their combination potential with anti-HER2 agents. Palbociclib and ribociclib are primarily metabolized by CYP3A4, necessitating careful attention to concomitant medications that may affect drug levels [111]. Abemaciclib has a more complex metabolism involving multiple cytochrome enzymes and active metabolites [111].

Drug interaction studies have not identified clinically significant interactions between CDK4/6 inhibitors and standard anti-HER2 agents including trastuzumab and pertuzumab [111]. However, interactions with tyrosine kinase inhibitors such as lapatinib and neratinib require further investigation [49,50].

### 8.3. Monitoring and Dose Management Guidelines

Effective management of CDK4/6 inhibitor combinations requires systematic monitoring protocols and standardized dose modification algorithms [112]. Complete blood counts should be obtained at baseline, every two weeks for the first two months, then monthly thereafter. More frequent monitoring may be necessary during the first cycle or following dose modifications [24,26,113].

Dose reduction strategies follow established protocols developed for each CDK4/6 inhibitor. For palbociclib, the standard starting dose is 125 mg daily with reductions to 100 mg and 75 mg for grade 3/4 toxicities [24]. Ribociclib follows a similar pattern with starting doses of 600 mg daily [113]. Abemaciclib utilizes a continuous dosing schedule at 150 mg twice daily with reductions to 100 mg and 50 mg twice daily [26].

Treatment interruption guidelines recommend holding CDK4/6 inhibitors for grade 3/4 hematologic toxicities until recovery to grade 1 or baseline [114]. Anti-HER2 therapy can typically continue during CDK4/6 inhibitor interruptions, maintaining disease control while allowing hematologic recovery.

## 9. Emerging Therapeutics and Future Directions

### 9.1. Novel CDK4/6 Inhibitors

Several next-generation CDK4/6 inhibitors are advancing through clinical development with potential advantages over existing agents. Dalpiciclib (SHR6390) has received approval in China for HR+/HER2- advanced breast cancer and is now being investigated in HER2+ combinations [34,71,72,111]. The agent demonstrates comparable efficacy to established CDK4/6 inhibitors with a potentially improved gastrointestinal toxicity profile [112,113].

TQB3616 represents another promising agent with preclinical evidence suggesting superior anti-tumor activity compared to abemaciclib. At the highest concentrations tested, TQB3616 completely inhibited breast cancer cell proliferation and demonstrated enhanced activity in combination with both fulvestrant and trastuzumab [114]. Phase II and III trials are planned across multiple settings (NCT05365178, NCT05780567, NCT04796623).

FCN-437c has emerged as a highly selective CDK4/6 inhibitor with minimal activity against CDK1, CDK2, or CDK5. Phase Ib trials combining FCN-437c with letrozole demonstrated promising anti-tumor activity with progression-free survival rates of 92.3% and 83.5% at 6 and 12 months, respectively (NCT04488107). The agent is now advancing to phase III evaluation (NCT05438810 and NCT05439499).

### 9.2. CDK7 Inhibition and Beyond

The recognition that CDK7 serves as a master regulator of both transcriptional CDKs and cell cycle CDKs has opened new therapeutic avenues [115]. SY-1365, a potent and selective CDK7 inhibitor, is currently undergoing phase I evaluation in solid tumors including breast cancer (NCT03134638).

CDK7 inhibition offers potential advantages over CDK4/6 targeting by simultaneously disrupting both cell cycle progression and transcriptional regulation. The agent demonstrates particular promise in estrogen-receptor-positive breast cancer through its ability to disrupt ER transcriptional activity. Combination studies with selective estrogen receptor degraders (SERDs) have shown synergistic activity in preclinical models [75].

### 9.3. Triple Combination Strategies

The integration of three targeted agents—anti-HER2 therapy, CDK4/6 inhibitors, and endocrine therapy—represents a promising strategy for optimizing outcomes in HER2+/HR+ breast cancer.

The TOUCH trial is evaluating ribociclib plus trastuzumab, pertuzumab, and letrozole in elderly patients with ER+/HER2+ primary breast cancer. This randomized phase II neoadjuvant study aims to explore biomarker-driven treatment selection using RB signature analysis (NCT03644186) (Table 3).

Novel anti-HER2 agents are also being incorporated into triple or quadruple combinations. Ongoing trials include the UCLA B-13 (NCT05319873), a phase Ib/II trial investigating the combination of trastuzumab and tucatinib with ribociclib and fulvestrant in the neoadjuvant setting, and the NCT05800756 trial, combining pyrotinib and trastuzumab with dalpiciclib and letrozole (Table 3).

### 9.4. Immunotherapy Integration

The potential for combining CDK4/6 inhibitors with immune checkpoint inhibitors in HER2+ breast cancer remains an area of active investigation. Preclinical studies have suggested that CDK4/6 inhibition may enhance immune system activation through effects on T-cell function and tumor antigen presentation [115].

However, clinical trials combining CDK4/6 inhibitors with immune checkpoint inhibitors have yielded mixed results in breast cancer [116,117,118,119]. Some trial investigating pembrolizumab plus CDK4/6 inhibitor therapy in HR+ breast cancer was terminated early due to lack of efficacy [120]. Nevertheless, the unique immune microenvironment of HER2+ tumors may provide different opportunities for combination approaches [121,122].

### 9.5. Precision Medicine Applications

The future of CDK4/6 inhibitor therapy in HER2+/HR+ breast cancer will likely depend on the development of robust predictive biomarkers and precision medicine approaches. Advances in genomic sequencing, transcriptomic profiling, and artificial intelligence are enabling more sophisticated patient selection strategies.

Machine learning approaches are being applied to identify complex biomarker signatures that predict CDK4/6 inhibitor response. These methods may identify previously unrecognized patterns in genomic, transcriptomic, and clinical data that improve patient selection [123].

Real-world evidence studies are providing valuable insights into the effectiveness of CDK4/6 inhibitor combinations outside of clinical trial populations. These studies help identify patient populations that may benefit from treatment despite not meeting clinical trial eligibility criteria [124].

## 10. Clinical Practice Implications and Treatment Algorithms

### 10.1. Current Treatment Recommendations

Based on the accumulating clinical evidence, CDK4/6 inhibitors are increasingly being incorporated into treatment guidelines for HER2+/HR+ breast cancer. The NCCN Clinical Practice Guidelines in Oncology (NCCN Guidelines^®^ V.4.2025. – April 17, 2025) now include CDK4/6 inhibitor combinations as preferred regimens for HER2+/HR+ metastatic breast cancer patients following initial chemotherapy and anti-HER2 therapy [125].

The European Society for Medical Oncology (ESMO) guidelines have similarly evolved to recognize the role of CDK4/6 inhibitors in this population. The strong evidence from the PATINA trial has led to the inclusion of palbociclib plus anti-HER2 therapy and endocrine therapy as a standard maintenance approach [116,117].

Treatment selection should consider multiple factors including performance status, comorbidities, prior therapies, and molecular characteristics. Patients with luminal A or B PAM50 subtypes appear to derive particular benefit, though subtype testing is not yet routinely available in all clinical settings.

### 10.2. Treatment Sequencing Considerations

The optimal sequencing of CDK4/6 inhibitors within the broader treatment paradigm for HER2+/HR+ breast cancer remains an evolving area. In the metastatic setting, the PATINA trial design suggests that CDK4/6 inhibitors are most effective when used as maintenance therapy following initial disease control with chemotherapy and anti-HER2 therapy [32].

However, the role of CDK4/6 inhibitors in first-line therapy remains to be fully established. Ongoing trials are investigating upfront combinations with anti-HER2 therapy and endocrine therapy, potentially avoiding the need for initial chemotherapy in selected patients (NCT05969184, NCT03772353).

The integration of T-DXd into treatment algorithms presents additional complexity. Given the remarkable efficacy of T-DXd in HER2+ breast cancer, its optimal positioning relative to CDK4/6 inhibitor therapy requires careful consideration. Sequential approaches utilizing both strategies may provide the greatest long-term benefit.

### 10.3. Special Patient Populations

Several special patient populations require individualized approaches to CDK4/6 inhibitor therapy. Elderly patients may experience increased toxicity from combination therapy but can derive similar efficacy benefits when appropriately managed. The TOUCH trial specifically addresses this population with modified dosing and monitoring approaches (NCT03644186).

Patients with brain metastases represent another important consideration. While traditional CDK4/6 inhibitors have limited central nervous system penetration, newer agents including dalpiciclib and combinations with CNS-active anti-HER2 agents may provide improved outcomes [118,119].

Young patients with concerns about fertility require careful counseling regarding the potential effects of combination therapy on reproductive function. The reversible nature of CDK4/6 inhibitor-induced amenorrhea should be discussed, though long-term fertility outcomes require further study [120].

## 11. Economic Considerations and Global Access

### 11.1. Cost-Effectiveness Analysis

The economic impact of CDK4/6 inhibitor therapy in HER2+/HR+ breast cancer represents an important consideration for healthcare systems worldwide. While these agents significantly improve clinical outcomes, their high cost requires careful economic evaluation [121].

Cost-effectiveness analyses from multiple healthcare systems have generally supported the use of CDK4/6 inhibitors in HR+/HER2- breast cancer [122]. However, specific economic evaluations in the HER2+ population are limited, and the cost-effectiveness profile may differ given the availability of alternative effective therapies.

The duration of treatment represents a key economic factor, with some patients receiving CDK4/6 inhibitors for extended periods. Value-based pricing approaches that consider treatment duration and outcomes may help improve access while managing costs [122].

### 11.2. Global Access and Healthcare Disparities

Access to CDK4/6 inhibitors varies significantly across different healthcare systems and geographic regions. In many low- and middle-income countries, access remains limited due to cost considerations and regulatory barriers [123].

The development of biosimilar CDK4/6 inhibitors and generic formulations may improve global access in the future. China’s approval of dalpiciclib represents an important step toward expanding options and potentially reducing costs [124].

International cooperation and assistance programs may help improve access to CDK4/6 inhibitors in resource-limited settings. The development of treatment algorithms that optimize outcomes while considering resource constraints will be essential.

## 12. Conclusions

The integration of CDK4/6 inhibitors into the treatment paradigm for HER2+/HR+ breast cancer represents a significant advancement that addresses the unique biological characteristics and clinical challenges of this distinct breast cancer subtype. The convergence of HER2 and estrogen receptor signaling on cell cycle regulatory mechanisms provides compelling biological rationale for targeting CDK4/6, while mounting clinical evidence demonstrates meaningful improvements in patient outcomes across multiple disease settings.

The phase III PATINA trial has established a new standard of care in the metastatic setting, with the combination of palbociclib plus anti-HER2 therapy and endocrine therapy delivering a clinically meaningful 15.2-month progression-free survival benefit. This represents one of the largest treatment effects observed in HER2+ breast cancer trials and validates the biological rationale for combination approaches. Supporting evidence from trials including MonarcHER, PATRICIA II, and DETECT V further confirms the therapeutic potential of CDK4/6 inhibition across different clinical contexts.

In the neoadjuvant setting, emerging evidence from trials such as NA-PHER2 and MUKDEN-01 suggests that CDK4/6 inhibitor combinations may enable chemotherapy de-escalation strategies while maintaining or improving clinical outcomes. The dramatic antiproliferative effects demonstrated through Ki67 suppression, combined with encouraging pathologic response rates, support the continued investigation of non-chemotherapy approaches for selected patients with HER2+/HR+ early breast cancer. The emergence of novel CDK inhibitors including dalpiciclib, TQB3616, and CDK7-targeted agents expands the therapeutic landscape and provides opportunities for improved efficacy and tolerability profiles. Triple combination strategies incorporating anti-HER2 therapy, CDK4/6 inhibitors, and endocrine therapy show particular promise but require careful safety evaluation and patient selection.

Despite these advances, several critical limitations of the current evidence base warrant acknowledgment. First, while the PATINA trial provides the strongest level of evidence for CDK4/6 inhibitor use in this population, it employed PFS as the primary endpoint, and mature overall survival data are still awaited. Second, substantial heterogeneity exists across the included trials in terms of patient selection criteria, prior treatment lines, endocrine therapy partners, and biomarker definitions, limiting direct cross-trial comparisons and the ability to define a single optimal treatment algorithm. Third, the absence of validated predictive biomarkers means that treatment decisions currently rely on clinical factors rather than biology-driven selection. Fourth, much of the available data, particularly in the neoadjuvant setting, derives from single-arm phase II studies with limited sample sizes, restricting conclusions about comparative efficacy. As is typical of scoping reviews, the quality or risk of bias of included studies was not formally assessed, limiting conclusions about the robustness of the evidence, particularly from early-phase or non-randomized trials. Although the search was comprehensive, some relevant studies may have been missed due to publication bias, English-language restrictions, and the rapid evolution of the HER2+/HR+ treatment landscape. Heterogeneity across trials—including differences in patient populations, therapeutic backbones, endocrine partners, biomarker assessments, and clinical endpoints—limits direct comparability and the ability to define optimal treatment strategies. Additionally, many available data come from small or early-phase studies with limited follow-up, restricting conclusions about long-term outcomes, sequencing considerations, and real-world applicability.

Looking forward, the field is poised for continued advancement through several key areas. Precision medicine approaches utilizing advanced genomic and transcriptomic analyses may enable more sophisticated patient selection and treatment optimization. Real-world evidence studies will provide valuable insights into treatment effectiveness across diverse patient populations and healthcare settings. The development of resistance biomarkers and monitoring strategies may help optimize treatment duration and sequencing decisions. Key priorities for future research include:

(1) Prospective validation of predictive biomarkers such as PAM50 subtyping and CCNE1/RB1 expression ratios in large-scale registrational trials;
(2) Randomized studies directly comparing the sequencing of CDK4/6 inhibitors versus T-DXd in first and later lines of maintenance therapy;
(3) Investigation of combination strategies incorporating next-generation agents like bispecific antibodies (zanidatamab) and novel ADCs;
(4) Trials specifically designed for chemotherapy de-escalation in the neoadjuvant setting with appropriate long-term survival endpoints.

The integration of artificial intelligence-driven algorithms for treatment selection and the expansion of liquid biopsy-based monitoring represent additional frontiers that may accelerate progress in this field.

The successful use of CDK4/6 inhibitors in HER2+/HR+ breast cancer treatment paradigms exemplifies the potential for targeted therapy combinations to address complex tumor biology through rational drug development. As our understanding of resistance mechanisms and predictive biomarkers continues to evolve, the goal of personalized treatment approaches that maximize efficacy while minimizing toxicity becomes increasingly achievable. This progress ultimately translates to improved outcomes and quality of life for patients with this challenging breast cancer subtype.

Finally, this review aimed to map existing evidence rather than provide quantitative estimates of effect, and the conclusions should be interpreted within this descriptive framework.

## Data Availability

No new data were created or analyzed in this study. Data sharing is not applicable to this article.

## Supplementary Materials

The following supporting information can be downloaded at: https://www.mdpi.com/article/10.3390/jpm16030143/s1: Preferred Reporting Items for Systematic reviews and Meta-Analyses extension for Scoping Reviews (PRISMA-ScR) Checklist. Reference [126] is cited in the Supplementary Materials.

## References

[1] Godoy-Ortiz A., Sanchez-Muñoz A., Chica Parrado M.R., Álvarez M., Ribelles N., Rueda Dominguez A., Alba E. (2019). Deciphering HER2 Breast Cancer Disease: Biological and Clinical Implications. Front. Oncol..

[2] Sung H., Ferlay J., Siegel R.L., Laversanne M., Soerjomataram I., Jemal A., Bray F. (2021). Global Cancer Statistics 2020: GLOBOCAN Estimates of Incidence and Mortality Worldwide for 36 Cancers in 185 Countries. CA A Cancer J. Clin..

[3] Gion M., Trapani D., Cortés A., Valenza C., Lin N., Cortés J., Curigliano G. (2022). Systemic Therapy for HER2-Positive Metastatic Breast Cancer: Moving Into a New Era. Am. Soc. Clin. Oncol. Educ. Book.

[4] Baselga J., Perez E.A., Pienkowski T., Bell R. (2006). Adjuvant Trastuzumab: A Milestone in the Treatment of HER-2-Positive Early Breast Cancer. Oncologist.

[5] Hudis C.A. (2007). Trastuzumab—Mechanism of Action and Use in Clinical Practice. N. Engl. J. Med..

[6] Labidi S., Mejri N., Lagha A., Daoud N., El Benna H., Afrit M., Boussen H. (2016). Targeted Therapies in HER2-Overexpressing Metastatic Breast Cancer. Breast Care.

[7] Swain S.M., Shastry M., Hamilton E. (2023). Targeting HER2-Positive Breast Cancer: Advances and Future Directions. Nat. Rev. Drug Discov..

[8] Prat A., Carey L.A., Adamo B., Vidal M., Tabernero J., Cortés J., Parker J.S., Perou C.M., Baselga J. (2014). Molecular Features and Survival Outcomes of the Intrinsic Subtypes Within HER2-Positive Breast Cancer. J. Natl. Cancer Inst..

[9] Harbeck N. (2015). Insights into Biology of Luminal HER2 vs. Enriched HER2 Subtypes: Therapeutic Implications. Breast.

[10] Goldhirsch A., Winer E.P., Coates A.S., Gelber R.D., Piccart-Gebhart M., Thürlimann B., Senn H.-J., Albain K.S., André F., Bergh J. (2013). Personalizing the Treatment of Women with Early Breast Cancer: Highlights of the St Gallen International Expert Consensus on the Primary Therapy of Early Breast Cancer 2013. Ann. Oncol..

[11] Bianchini G., Prat A., Pickl M., Belousov A., Koehler A., Semiglazov V., Eiermann W., Tjulandin S., Biakhov M., Lluch A. (2011). Response to Neoadjuvant Trastuzumab and Chemotherapy in ER+ and ER− HER2-Positive Breast Cancers: Gene Expression Analysis. J. Clin. Oncol..

[12] Vici P., Pizzuti L., Natoli C., Gamucci T., Di Lauro L., Barba M., Sergi D., Botti C., Michelotti A., Moscetti L. (2015). Triple Positive Breast Cancer: A Distinct Subtype?. Cancer Treat. Rev..

[13] Nahta R., O’Regan R.M. (2012). Therapeutic Implications of Estrogen Receptor Signaling in HER2-Positive Breast Cancers. Breast Cancer Res. Treat..

[14] Xie J., Yang Z., Li Z., Zhang T., Chen H., Chen X., Dai Z., Chen T., Hou J. (2025). Triple-Positive Breast Cancer: Navigating Heterogeneity and Advancing Multimodal Therapies for Improving Patient Outcomes. Cancer Cell Int..

[15] Vernieri C., Milano M., Brambilla M., Mennitto A., Maggi C., Cona M.S., Prisciandaro M., Fabbroni C., Celio L., Mariani G. (2019). Resistance Mechanisms to Anti-HER2 Therapies in HER2-Positive Breast Cancer: Current Knowledge, New Research Directions and Therapeutic Perspectives. Crit. Rev. Oncol. Hematol..

[16] Arpino G., Ferrero J.-M., De La Haba-Rodriguez J., Easton V., Schuhmacher C., Restuccia E., Rimawi M. (2017). Abstract S3-04: Primary Analysis of PERTAIN: A Randomized, Two-Arm, Open-Label, Multicenter Phase II Trial Assessing the Efficacy and Safety of Pertuzumab given in Combination with Trastuzumab plus an Aromatase Inhibitor in First-Line Patients with HER2-Positive and Hormone Receptor-Positive Metastatic or Locally Advanced Breast Cancer. Cancer Res..

[17] Di Cosimo S., Arpino G., Generali D. (2014). Neoadjuvant Treatment of HER2 and Hormone-Receptor Positive Breast Cancer—Moving beyond Pathological Complete Response. Breast.

[18] Swain S.M., Baselga J., Kim S.-B., Ro J., Semiglazov V., Campone M., Ciruelos E., Ferrero J.-M., Schneeweiss A., Heeson S. (2015). Pertuzumab, Trastuzumab, and Docetaxel in HER2-Positive Metastatic Breast Cancer. N. Engl. J. Med..

[19] Goetz M.P., Gradishar W.J., Anderson B.O., Abraham J., Aft R., Allison K.H., Blair S.L., Burstein H.J., Dang C., Elias A.D. (2019). Breast Cancer, Version 3.2018. J. Natl. Compr. Cancer Netw..

[20] Goel S., Wang Q., Watt A.C., Tolaney S.M., Dillon D.A., Li W., Ramm S., Palmer A.C., Yuzugullu H., Varadan V. (2016). Overcoming Therapeutic Resistance in HER2-Positive Breast Cancers with CDK4/6 Inhibitors. Cancer Cell.

[21] Malumbres M. (2016). CDK4/6 Inhibitors resTORe Therapeutic Sensitivity in HER2 + Breast Cancer. Cancer Cell.

[22] O’Leary B., Finn R.S., Turner N.C. (2016). Treating Cancer with Selective CDK4/6 Inhibitors. Nat. Rev. Clin. Oncol..

[23] Goel S., Bergholz J.S., Zhao J.J. (2022). Targeting CDK4 and CDK6 in Cancer. Nat. Rev. Cancer.

[24] Finn R.S., Martin M., Rugo H.S., Jones S., Im S.-A., Gelmon K., Harbeck N., Lipatov O.N., Walshe J.M., Moulder S. (2016). Palbociclib and Letrozole in Advanced Breast Cancer. N. Engl. J. Med..

[25] Hortobagyi G.N., Stemmer S.M., Burris H.A., Yap Y.-S., Sonke G.S., Hart L., Campone M., Petrakova K., Winer E.P., Janni W. (2022). Overall Survival with Ribociclib plus Letrozole in Advanced Breast Cancer. N. Engl. J. Med..

[26] Goetz M.P., Toi M., Campone M., Sohn J., Paluch-Shimon S., Huober J., Park I.H., Trédan O., Chen S.-C., Manso L. (2017). MONARCH 3: Abemaciclib As Initial Therapy for Advanced Breast Cancer. J. Clin. Oncol..

[27] Spring L.M., Wander S.A., Andre F., Moy B., Turner N.C., Bardia A. (2020). Cyclin-Dependent Kinase 4 and 6 Inhibitors for Hormone Receptor-Positive Breast Cancer: Past, Present, and Future. Lancet.

[28] Kitagawa M., Higashi H., Jung H.-K., Suzuki-Takahashi I., Ikeda M., Tamai K., Kato J., Segawa K., Yoshida E., Nishimura S. (1996). The Consensus Motif for Phosphorylation by Cyclin Dl-Cdk4 Is Different from That for Phosphorylation by Cyclin A/E-Cdk2. EMBO J..

[29] Nikolai B.C., Lanz R.B., York B., Dasgupta S., Mitsiades N., Creighton C.J., Tsimelzon A., Hilsenbeck S.G., Lonard D.M., Smith C.L. (2016). HER2 Signaling Drives DNA Anabolism and Proliferation through SRC-3 Phosphorylation and E2F1-Regulated Genes. Cancer Res..

[30] Zhang K., Hong R., Kaping L., Xu F., Xia W., Qin G., Zheng Q., Lu Q., Zhai Q., Shi Y. (2019). CDK4/6 Inhibitor Palbociclib Enhances the Effect of Pyrotinib in HER2-Positive Breast Cancer. Cancer Lett..

[31] Tolaney S.M., Goel S., Nadal J., Denys H., Borrego M.R., Litchfield L.M., Liu J., Appiah A.K., Chen Y., André F. (2024). Overall Survival and Exploratory Biomarker Analyses of Abemaciclib plus Trastuzumab with or without Fulvestrant versus Trastuzumab plus Chemotherapy in HR+, HER2+ Metastatic Breast Cancer Patients. Clin. Cancer Res..

[32] Loibl S., Metzger O., Mandrekar S.J., Mundhenke C., Seiler S., Valagussa P., DeMichele A., Lim E., Tripathy D., Winer E.P. (2018). PATINA: A Randomized, Open Label, Phase III Trial to Evaluate the Efficacy and Safety of Palbociclib + Anti-HER2 Therapy + Endocrine Therapy (ET) vs. Anti-HER2 Therapy + ET after Induction Treatment for Hormone Receptor Positive (HR+)/HER2-Positive Metastatic Breast Cancer (MBC). Ann. Oncol..

[33] Moasser M.M. (2007). The Oncogene HER2: Its Signaling and Transforming Functions and Its Role in Human Cancer Pathogenesis. Oncogene.

[34] Yan M., Niu L., Lv H., Zhang M., Wang J., Liu Z., Chen X., Lu Z., Zhang C., Zeng H. (2023). Dalpiciclib and Pyrotinib in Women with HER2-Positive Advanced Breast Cancer: A Single-Arm Phase II Trial. Nat. Commun..

[35] Slamon D.J., Clark G.M., Wong S.G., Levin W.J., Ullrich A., McGuire W.L. (1987). Human Breast Cancer: Correlation of Relapse and Survival with Amplification of the HER-2/*Neu* Oncogene. Science.

[36] Glaviano A., Foo A.S.C., Lam H.Y., Yap K.C.H., Jacot W., Jones R.H., Eng H., Nair M.G., Makvandi P., Geoerger B. (2023). PI3K/AKT/mTOR Signaling Transduction Pathway and Targeted Therapies in Cancer. Mol. Cancer.

[37] Cam H., Dynlacht B.D. (2003). Emerging Roles for E2F: Beyond the G1/S Transition and DNA Replication. Cancer Cell.

[38] Fuentes N., Silveyra P. (2019). Estrogen Receptor Signaling Mechanisms. Advances in Protein Chemistry and Structural Biology.

[39] Caldon C.E., Sergio C.M., Schütte J., Boersma M.N., Sutherland R.L., Carroll J.S., Musgrove E.A. (2009). Estrogen Regulation of Cyclin E2 Requires Cyclin D1 but Not C-Myc. Mol. Cell. Biol..

[40] Arpino G., Wiechmann L., Osborne C.K., Schiff R. (2008). Crosstalk between the Estrogen Receptor and the HER Tyrosine Kinase Receptor Family: Molecular Mechanism and Clinical Implications for Endocrine Therapy Resistance. Endocr. Rev..

[41] Pegram M., Jackisch C., Johnston S.R.D. (2023). Estrogen/HER2 Receptor Crosstalk in Breast Cancer: Combination Therapies to Improve Outcomes for Patients with Hormone Receptor-Positive/HER2-Positive Breast Cancer. npj Breast Cancer.

[42] Topacio B.R., Zatulovskiy E., Cristea S., Xie S., Tambo C.S., Rubin S.M., Sage J., Kõivomägi M., Skotheim J.M. (2019). Cyclin D-Cdk4,6 Drives Cell-Cycle Progression via the Retinoblastoma Protein’s C-Terminal Helix. Mol. Cell.

[43] Asghar U., Witkiewicz A.K., Turner N.C., Knudsen E.S. (2015). The History and Future of Targeting Cyclin-Dependent Kinases in Cancer Therapy. Nat. Rev. Drug Discov..

[44] Glaviano A., Wander S.A., Baird R.D., Yap K.C.-H., Lam H.Y., Toi M., Carbone D., Geoerger B., Serra V., Jones R.H. (2024). Mechanisms of Sensitivity and Resistance to CDK4/CDK6 Inhibitors in Hormone Receptor-Positive Breast Cancer Treatment. Drug Resist. Updates.

[45] Rimawi M.F., De Angelis C., Schiff R. (2015). Resistance to Anti-HER2 Therapies in Breast Cancer. Am. Soc. Clin. Oncol. Educ. Book.

[46] Finn R.S., Dering J., Conklin D., Kalous O., Cohen D.J., Desai A.J., Ginther C., Atefi M., Chen I., Fowst C. (2009). PD 0332991, a Selective Cyclin D Kinase 4/6 Inhibitor, Preferentially Inhibits Proliferation of Luminal Estrogen Receptor-Positive Human Breast Cancer Cell Lines in Vitro. Breast Cancer Res..

[47] O’Brien N., Conklin D., Beckmann R., Luo T., Chau K., Thomas J., Mc Nulty A., Marchal C., Kalous O., Von Euw E. (2018). Preclinical Activity of Abemaciclib Alone or in Combination with Antimitotic and Targeted Therapies in Breast Cancer. Mol. Cancer Ther..

[48] Dean J.L., McClendon A.K., Hickey T.E., Butler L.M., Tilley W.D., Witkiewicz A.K., Knudsen E.S. (2012). Therapeutic Response to CDK4/6 Inhibition in Breast Cancer Defined by Ex Vivo Analyses of Human Tumors. Cell Cycle.

[49] Witkiewicz A.K., Cox D., Knudsen E.S. (2014). CDK4/6 Inhibition Provides a Potent Adjunct to Her2-Targeted Therapies in Preclinical Breast Cancer Models. Genes Cancer.

[50] Zhao M., Scott S., Evans K.W., Yuca E., Saridogan T., Zheng X., Wang H., Korkut A., Cruz Pico C.X., Demirhan M. (2021). Combining Neratinib with CDK4/6, mTOR, and MEK Inhibitors in Models of HER2-Positive Cancer. Clin. Cancer Res..

[51] ElChaarani B., Stires H., Pohlmann P.R., Riggins R. (2017). Pre-Clinical Analysis of the CDK4/6 Inhibitor Palbociclib in HER2-Positive Breast Cancer. J. Clin. Oncol..

[52] Brasó-Maristany F., Griguolo G., Pascual T., Paré L., Nuciforo P., Llombart-Cussac A., Bermejo B., Oliveira M., Morales S., Martínez N. (2020). Phenotypic Changes of HER2-Positive Breast Cancer during and after Dual HER2 Blockade. Nat. Commun..

[53] Sinclair W.D., Cui X. (2022). The Effects of HER2 on CDK4/6 Activity in Breast Cancer. Clin. Breast Cancer.

[54] O’Sullivan C.C., Suman V.J., Goetz M.P. (2019). The Emerging Role of CDK4/6i in HER2-Positive Breast Cancer. Ther. Adv. Med. Oncol..

[55] Zhang C., Zhou F., Zou J., Fang Y., Liu Y., Li L., Hou J., Wang G., Wang H., Lai X. (2024). Clinical Considerations of CDK4/6 Inhibitors in HER2 Positive Breast Cancer. Front. Oncol..

[56] Zhang J., Meng Y., Wang B., Wang L., Cao J., Tao Z., Li T., Yao W., Hu X. (2022). Dalpiciclib Combined with Pyrotinib and Letrozole in Women with HER2-Positive, Hormone Receptor-Positive Metastatic Breast Cancer (LORDSHIPS): A Phase Ib Study. Front. Oncol..

[57] Ciruelos E., Villagrasa P., Pascual T., Oliveira M., Pernas S., Paré L., Escrivá-de-Romaní S., Manso L., Adamo B., Martínez E. (2020). Palbociclib and Trastuzumab in HER2-Positive Advanced Breast Cancer: Results from the Phase II SOLTI-1303 PATRICIA Trial. Clin. Cancer Res..

[58] Tolaney S.M., Wardley A.M., Zambelli S., Hilton J.F., Troso-Sandoval T.A., Ricci F., Im S.-A., Kim S.-B., Johnston S.R., Chan A. (2020). Abemaciclib plus Trastuzumab with or without Fulvestrant versus Trastuzumab plus Standard-of-Care Chemotherapy in Women with Hormone Receptor-Positive, HER2-Positive Advanced Breast Cancer (monarcHER): A Randomised, Open-Label, Phase 2 Trial. Lancet Oncol..

[59] Wang X., Zhao S., Xin Q., Zhang Y., Wang K., Li M. (2024). Recent Progress of CDK4/6 Inhibitors’ Current Practice in Breast Cancer. Cancer Gene Ther..

[60] Janni W., Fehm T.N., Mueller V., De Gregorio A.M.B., Decker T., Hartkopf A.D., Just M., Sagasser J., Schmidt M., Wimberger P. (2024). 350MO Omission of Chemotherapy and Addition of the CDK4/6 Inhibitor Ribociclib in HER2-Positive and Hormone-Receptor Positive Metastatic Breast Cancer—Second Interim Efficacy Analysis of the Randomized Phase III DETECT V Trial. Ann. Oncol..

[61] Goel S., Pernas S., Tan-Wasielewski Z., Barry W.T., Bardia A., Rees R., Andrews C., Tahara R.K., Trippa L., Mayer E.L. (2019). Ribociclib Plus Trastuzumab in Advanced HER2-Positive Breast Cancer: Results of a Phase 1b/2 Trial. Clin. Breast Cancer.

[62] Haley B., Batra K., Sahoo S., Froehlich T., Klemow D., Unni N., Ahn C., Rodriguez M., Hullings M., Frankel A.E. (2021). A Phase I/Ib Trial of PD 0332991 (Palbociclib) and T-DM1 in HER2-Positive Advanced Breast Cancer After Trastuzumab and Taxane Therapy. Clin. Breast Cancer.

[63] Escrivá-de-Romani S., Cejalvo J.M., Alba E., Friedmann J., Rodríguez-Lescure Á., Savard M.-F., Pezo R.C., Gion M., Ruiz-Borrego M., Hamilton E. (2025). Zanidatamab plus Palbociclib and Fulvestrant in Previously Treated Patients with Hormone Receptor-Positive, HER2-Positive Metastatic Breast Cancer: Primary Results from a Two-Part, Multicentre, Single-Arm, Phase 2a Study. Lancet Oncol..

[64] Schettini F., Pascual T., Conte B., Chic N., Brasó-Maristany F., Galván P., Martínez O., Adamo B., Vidal M., Muñoz M. (2020). HER2-Enriched Subtype and Pathological Complete Response in HER2-Positive Breast Cancer: A Systematic Review and Meta-Analysis. Cancer Treat. Rev..

[65] Gianni L., Bisagni G., Colleoni M., Del Mastro L., Zamagni C., Mansutti M., Zambetti M., Frassoldati A., De Fato R., Valagussa P. (2018). Neoadjuvant Treatment with Trastuzumab and Pertuzumab plus Palbociclib and Fulvestrant in HER2-Positive, ER-Positive Breast Cancer (NA-PHER2): An Exploratory, Open-Label, Phase 2 Study. Lancet Oncol..

[66] De Azambuja E., Holmes A.P., Piccart-Gebhart M., Holmes E., Di Cosimo S., Swaby R.F., Untch M., Jackisch C., Lang I., Smith I. (2014). Lapatinib with Trastuzumab for HER2-Positive Early Breast Cancer (NeoALTTO): Survival Outcomes of a Randomised, Open-Label, Multicentre, Phase 3 Trial and Their Association with Pathological Complete Response. Lancet Oncol..

[67] Rimawi M.F., Mayer I.A., Forero A., Nanda R., Goetz M.P., Rodriguez A.A., Pavlick A.C., Wang T., Hilsenbeck S.G., Gutierrez C. (2013). Multicenter Phase II Study of Neoadjuvant Lapatinib and Trastuzumab with Hormonal Therapy and Without Chemotherapy in Patients with Human Epidermal Growth Factor Receptor 2–Overexpressing Breast Cancer: TBCRC 006. J. Clin. Oncol..

[68] Harbeck N. (2018). Neoadjuvant Treatment of HER2-Positive Breast Cancer: Should Therapy Differ Based on Hormone Receptor Status?. Ther. Adv. Med. Oncol..

[69] Pérez-García J.M., Cortés J., Ruiz-Borrego M., Colleoni M., Stradella A., Bermejo B., Dalenc F., Escrivá-de-Romaní S., Calvo Martínez L., Ribelles N. (2024). 3-Year Invasive Disease-Free Survival with Chemotherapy de-Escalation Using an 18F-FDG-PET-Based, Pathological Complete Response-Adapted Strategy in HER2-Positive Early Breast Cancer (PHERGain): A Randomised, Open-Label, Phase 2 Trial. Lancet.

[70] Liu S., Yu M., Mou E., Wang M., Liu S., Xia L., Li H., Tang H., Feng Y., Yu X. (2025). The Optimal Neoadjuvant Treatment Strategy for HR+/HER2 + Breast Cancer: A Network Meta-Analysis. Sci. Rep..

[71] Yang Z.-J., Xin F., Chen Z.-J., Yu Y., Wang X., Cao X.-C. (2024). Real-World Data on Neoadjuvant Chemotherapy with Dual-Anti HER2 Therapy in HER2 Positive Breast Cancer. BMC Cancer.

[72] Loft M., Lok S.W., De Boer R., Malik L., Greenberg S., Yeo B., Anton A., Nottage M., Wong V., Nott L. (2023). Addition of Endocrine Therapy to Dual Anti-HER2 Targeted Therapy in Initial Treatment of HER2 + /HR + Metastatic Breast Cancer. Breast Cancer Res. Treat..

[73] Ademuyiwa F.O., Northfelt D.W., O’Connor T., Levine E., Luo J., Tao Y., Hoog J., Laury M.L., Summa T., Hammerschmidt T. (2023). A Phase II Study of Palbociclib plus Letrozole plus Trastuzumab as Neoadjuvant Treatment for Clinical Stages II and III ER+ HER2+ Breast Cancer (PALTAN). npj Breast Cancer.

[74] Niu N., Qiu F., Xu Q., He G., Gu X., Guo W., Zhang D., Li Z., Zhao Y., Li Y. (2022). A Multicentre Single Arm Phase 2 Trial of Neoadjuvant Pyrotinib and Letrozole plus Dalpiciclib for Triple-Positive Breast Cancer. Nat. Commun..

[75] Malorni L., Tyekucheva S., Gombos A., Hasler-Strub U., Zamagni C., Chakiba-Brugère C., Colleoni M., Mueller A., Minisini A.M., Taylor D. (2026). Palbociclib plus Letrozole versus Weekly Paclitaxel, Both in Combination with Trastuzumab plus Pertuzumab, as Neoadjuvant Treatment for Patients with HR+/HER2+ Early Breast Cancer: Primary Results from the Randomized Phase II TOUCH Trial (IBCSG 55-17). Ann. Oncol..

[76] Huo S., Xue J., Wang S., Shan H., Chen G., Niu N., Wang Y., Qiu F., Zhao Y., Xing F. (2024). A Pilot Trial of Neoadjuvant Pyrotinib plus Trastuzumab, Dalpiciclib, and Letrozole for Triple-positive Breast Cancer. MedComm.

[77] Liu C., Sun L., Niu N., Hou P., Chen G., Wang H., Zhang Z., Jiang X., Xu Q., Zhao Y. (2025). Molecular Classification of Hormone Receptor-Positive /HER2-Positive Breast Cancer Reveals Potential Neoadjuvant Therapeutic Strategies. Signal Transduct. Target. Ther..

[78] Wu J., Jiang Z., Liu Z., Yang B., Yang H., Tang J., Wang K., Liu Y., Wang H., Fu P. (2022). Neoadjuvant Pyrotinib, Trastuzumab, and Docetaxel for HER2-Positive Breast Cancer (PHEDRA): A Double-Blind, Randomized Phase 3 Trial. BMC Med..

[79] Krasniqi E., Goeman F., Pulito C., Palcau A.C., Ciuffreda L., Di Lisa F.S., Filomeno L., Barba M., Pizzuti L., Cappuzzo F. (2022). Biomarkers of Response and Resistance to CDK4/6 Inhibitors in Breast Cancer: Hints from Liquid Biopsy and microRNA Exploration. Int. J. Mol. Sci..

[80] Zhu Z., Turner N.C., Loi S., André F., Martin M., Diéras V., Gelmon K.A., Harbeck N., Zhang C., Cao J.Q. (2022). Comparative Biomarker Analysis of PALOMA-2/3 Trials for Palbociclib. npj Precis. Oncol..

[81] Asghar U.S., Kanani R., Roylance R., Mittnacht S. (2022). Systematic Review of Molecular Biomarkers Predictive of Resistance to CDK4/6 Inhibition in Metastatic Breast Cancer. JCO Precis. Oncol..

[82] Zhou F.H., Downton T., Freelander A., Hurwitz J., Caldon C.E., Lim E. (2023). CDK4/6 Inhibitor Resistance in Estrogen Receptor Positive Breast Cancer, a 2023 Perspective. Front. Cell Dev. Biol..

[83] Guarducci C., Bonechi M., Benelli M., Biagioni C., Boccalini G., Romagnoli D., Verardo R., Schiff R., Osborne C.K., De Angelis C. (2018). Cyclin E1 and Rb Modulation as Common Events at Time of Resistance to Palbociclib in Hormone Receptor-Positive Breast Cancer. npj Breast Cancer.

[84] Ma C.X., Gao F., Luo J., Northfelt D.W., Goetz M., Forero A., Hoog J., Naughton M., Ademuyiwa F., Suresh R. (2017). NeoPalAna: Neoadjuvant Palbociclib, a Cyclin-Dependent Kinase 4/6 Inhibitor, and Anastrozole for Clinical Stage 2 or 3 Estrogen Receptor–Positive Breast Cancer. Clin. Cancer Res..

[85] Reinhardt K., Stückrath K., Hartung C., Kaufhold S., Uleer C., Hanf V., Lantzsch T., Peschel S., John J., Pöhler M. (2022). PIK3CA-Mutations in Breast Cancer. Breast Cancer Res. Treat..

[86] Baselga J., Cortés J., Im S.-A., Clark E., Ross G., Kiermaier A., Swain S.M. (2014). Biomarker Analyses in CLEOPATRA: A Phase III, Placebo-Controlled Study of Pertuzumab in Human Epidermal Growth Factor Receptor 2–Positive, First-Line Metastatic Breast Cancer. J. Clin. Oncol..

[87] Seo Y., Park Y.H., Ahn J.S., Im Y.-H., Nam S.J., Cho S.Y., Cho E.Y. (2018). *PIK3CA* Mutations and Neoadjuvant Therapy Outcome in Patients with Human Epidermal Growth Factor Receptor 2-Positive Breast Cancer: A Sequential Analysis. J. Breast Cancer.

[88] Loibl S., Von Minckwitz G., Schneeweiss A., Paepke S., Lehmann A., Rezai M., Zahm D.M., Sinn P., Khandan F., Eidtmann H. (2014). *PIK3CA* Mutations Are Associated with Lower Rates of Pathologic Complete Response to Anti–Human Epidermal Growth Factor Receptor 2 (HER2) Therapy in Primary HER2-Overexpressing Breast Cancer. J. Clin. Oncol..

[89] Majewski I.J., Nuciforo P., Mittempergher L., Bosma A.J., Eidtmann H., Holmes E., Sotiriou C., Fumagalli D., Jimenez J., Aura C. (2015). *PIK3CA* Mutations Are Associated with Decreased Benefit to Neoadjuvant Human Epidermal Growth Factor Receptor 2–Targeted Therapies in Breast Cancer. J. Clin. Oncol..

[90] Guarneri V., Dieci M.V., Frassoldati A., Maiorana A., Ficarra G., Bettelli S., Tagliafico E., Bicciato S., Generali D.G., Cagossi K. (2015). Prospective Biomarker Analysis of the Randomized CHER-LOB Study Evaluating the Dual Anti-HER2 Treatment with Trastuzumab and Lapatinib Plus Chemotherapy as Neoadjuvant Therapy for HER2-Positive Breast Cancer. Oncologist.

[91] Takeshita T., Yamamoto Y., Yamamoto-Ibusuki M., Tomiguchi M., Sueta A., Murakami K., Iwase H. (2018). Clinical Significance of Plasma Cell-Free DNA Mutations in PIK3CA, AKT1, and ESR1 Gene According to Treatment Lines in ER-Positive Breast Cancer. Mol. Cancer.

[92] André F., Ciruelos E., Rubovszky G., Campone M., Loibl S., Rugo H.S., Iwata H., Conte P., Mayer I.A., Kaufman B. (2019). Alpelisib for *PIK3CA*-Mutated, Hormone Receptor–Positive Advanced Breast Cancer. N. Engl. J. Med..

[93] Raspé E., Coulonval K., Pita J.M., Paternot S., Rothé F., Twyffels L., Brohée S., Craciun L., Larsimont D., Kruys V. (2017). CDK 4 Phosphorylation Status and a Linked Gene Expression Profile Predict Sensitivity to Palbociclib. EMBO Mol. Med..

[94] Stover D.G., Coloff J.L., Barry W.T., Brugge J.S., Winer E.P., Selfors L.M. (2016). The Role of Proliferation in Determining Response to Neoadjuvant Chemotherapy in Breast Cancer: A Gene Expression–Based Meta-Analysis. Clin. Cancer Res..

[95] Shikanai A., Horimoto Y., Ishizuka Y., Uomori T., Nakai K., Arakawa A., Saito M. (2022). Clinicopathological Features Related to the Efficacy of CDK4/6 Inhibitor-Based Treatments in Metastatic Breast Cancer. Breast Cancer.

[96] Prat A., Bianchini G., Thomas M., Belousov A., Cheang M.C.U., Koehler A., Gómez P., Semiglazov V., Eiermann W., Tjulandin S. (2014). Research-Based PAM50 Subtype Predictor Identifies Higher Responses and Improved Survival Outcomes in HER2-Positive Breast Cancer in the NOAH Study. Clin. Cancer Res..

[97] Schoninger S.F., Blain S.W. (2020). The Ongoing Search for Biomarkers of CDK4/6 Inhibitor Responsiveness in Breast Cancer. Mol. Cancer Ther..

[98] DeMichele A., Clark A.S., Tan K.S., Heitjan D.F., Gramlich K., Gallagher M., Lal P., Feldman M., Zhang P., Colameco C. (2015). CDK 4/6 Inhibitor Palbociclib (PD0332991) in Rb+ Advanced Breast Cancer: Phase II Activity, Safety, and Predictive Biomarker Assessment. Clin. Cancer Res..

[99] Smith I., Robertson J., Kilburn L., Wilcox M., Evans A., Holcombe C., Horgan K., Kirwan C., Mallon E., Sibbering M. (2020). Long-Term Outcome and Prognostic Value of Ki67 after Perioperative Endocrine Therapy in Postmenopausal Women with Hormone-Sensitive Early Breast Cancer (POETIC): An Open-Label, Multicentre, Parallel-Group, Randomised, Phase 3 Trial. Lancet Oncol..

[100] Guarducci C., Bonechi M., Boccalini G., Benelli M., Risi E., Di Leo A., Malorni L., Migliaccio I. (2017). Mechanisms of Resistance to CDK4/6 Inhibitors in Breast Cancer and Potential Biomarkers of Response. Breast Care.

[101] Green A.R., Barros F.F.T., Abdel-Fatah T.M.A., Moseley P., Nolan C.C., Durham A.C., Rakha E.A., Chan S., Ellis I.O. (2014). HER2/HER3 Heterodimers and P21 Expression Are Capable of Predicting Adjuvant Trastuzumab Response in HER2+ Breast Cancer. Breast Cancer Res. Treat..

[102] Okutur K., Bassulu N., Dalar L., Aydin K., Bozkurt M., Pilanci K.N., Dogusoy G.B., Tecimer C., Mandel N.M., Demir G. (2015). Predictive and Prognostic Significance of P27, Akt, PTEN and PI3K Expression in HER2-Positive Metastatic Breast Cancer. Asian Pac. J. Cancer Prev..

[103] Nader-Marta G., Monteforte M., Agostinetto E., Cinquini M., Martins-Branco D., Langouo M., Llombart-Cusac A., Cortés J., Ignatiadis M., Torri V. (2024). Circulating Tumor DNA for Predicting Recurrence in Patients with Operable Breast Cancer: A Systematic Review and Meta-Analysis. ESMO Open.

[104] Zhong H.-J., Zhen Y., Chen S., Shi W., Liang X., Yang G.-J. (2025). Advances in CTC and ctDNA Detection Techniques: Opportunities for Improving Breast Cancer Care. Breast Cancer Res..

[105] Main S.C., Cescon D.W., Bratman S.V. (2022). Liquid Biopsies to Predict CDK4/6 Inhibitor Efficacy and Resistance in Breast Cancer. Cancer Drug Resist..

[106] De Placido P., Hughes M.E., Weipert C., Sammons S.L., Morganti S., Parsons H.A., Abravanel D., Giordano A., Smith K., Patel A. (2025). Use of Baseline Plasma Circulating Tumor DNA (ctDNA) to Predict Duration of Endocrine Therapy (ET) and CDK4/6 Inhibitor (CDK4/6i) Therapy (Tx) and to Analyze Intrinsic vs Acquired Endocrine Resistance. J. Clin. Oncol..

[107] Warrior S., Jaber D.A., Heater N.K., Marini S., Ma Y., Sun Z., Liu H., Zhang Q., Zhang Y., Cristofanilli M. (2025). The Use of Circulating Tumor DNA (ctDNA) to Evaluate Need for Additional Targeted Therapies in HER2 Positive Metastatic Breast Cancer (MBC). J. Clin. Oncol..

[108] Cani A.K., Hayes D.F. (2024). Breast Cancer Circulating Tumor Cells: Current Clinical Applications and Future Prospects. Clin. Chem..

[109] González-Conde M., Yáñez C., Abuín C., Keup C., Lago-Lestón R., Aybar M., Pedrouzo L., Palacios P., Curiel T., Cueva J. (2025). Gene Expression Analysis in Circulating Tumour Cells to Determine Resistance to CDK4/6 Inhibitors plus Endocrine Therapy in HR+/HER2− Metastatic Breast Cancer Patients. J. Transl. Med..

[110] Mastrantoni L., Orlandi A., Palazzo A., Garufi G., Fabi A., Daniele G., Giannarelli D., Tortora G., Bria E. (2023). The Likelihood of Being Helped or Harmed as a Patient-Centred Tool to Assess Cyclin Dependent Kinase 4/6 Inhibitors Clinical Impact and Safety in Metastatic Breast Cancer: A Systematic Review and Sensitivity-Analysis. eClinicalMedicine.

[111] Groenland S.L., Martínez-Chávez A., Van Dongen M.G.J., Beijnen J.H., Schinkel A.H., Huitema A.D.R., Steeghs N. (2020). Clinical Pharmacokinetics and Pharmacodynamics of the Cyclin-Dependent Kinase 4 and 6 Inhibitors Palbociclib, Ribociclib, and Abemaciclib. Clin. Pharmacokinet..

[112] Thill M., Schmidt M. (2018). Management of Adverse Events during Cyclin-Dependent Kinase 4/6 (CDK4/6) Inhibitor-Based Treatment in Breast Cancer. Ther. Adv. Med. Oncol..

[113] Hortobagyi G.N., Stemmer S.M., Burris H.A., Yap Y.-S., Sonke G.S., Paluch-Shimon S., Campone M., Blackwell K.L., André F., Winer E.P. (2016). Ribociclib as First-Line Therapy for HR-Positive, Advanced Breast Cancer. N. Engl. J. Med..

[114] Wekking D., Lambertini M., Dessì M., Denaro N., Bardanzellu F., Garrone O., Scartozzi M., Solinas C. (2023). CDK4/6 Inhibitors in the Treatment of Metastatic Breast Cancer: Focus on Toxicity and Safety. Semin. Oncol..

[115] Goel S., DeCristo M.J., Watt A.C., BrinJones H., Sceneay J., Li B.B., Khan N., Ubellacker J.M., Xie S., Metzger-Filho O. (2017). CDK4/6 Inhibition Triggers Anti-Tumour Immunity. Nature.

[116] Gennari A., André F., Barrios C.H., Cortés J., de Azambuja E., DeMichele A., Dent R., Fenlon D., Gligorov J., Hurvitz S.A. (2021). ESMO Clinical Practice Guideline for the Diagnosis, Staging and Treatment of Patients with Metastatic Breast Cancer. Ann. Oncol..

[117] Gennari A., Martins Branco D., Trapani D., Pentheroudakis G., Curigliano G., Harbeck N. ESMO Living Guideline: Metastatic Breast Cancer v1.2. https://www.esmo.org/guidelines/living-guidelines/esmo-living-guideline-metastatic-breast-cancer.

[118] Zhang P., Xu B., Gui L., Wang W., Xiu M., Zhang X., Sun G., Zhu X., Zou J. (2021). A Phase 1 Study of Dalpiciclib, a Cyclin-Dependent Kinase 4/6 Inhibitor in Chinese Patients with Advanced Breast Cancer. Biomark. Res..

[119] Luo J., Ren A., Si D., Yang J., Xu D., Li N. (2025). Drug Treatment of Breast Cancer Brain Metastases: Progress and Challenges. Discov. Oncol..

[120] Scavone G., Ottonello S., Blondeaux E., Arecco L., Scaruffi P., Stigliani S., Cardinali B., Borea R., Paudice M., Vellone V.G. (2023). The Role of Cyclin-Dependent Kinases (CDK) 4/6 in the Ovarian Tissue and the Possible Effects of Their Exogenous Inhibition. Cancers.

[121] Bhatt A., Nwosu J., Zhong L. (2023). EE55 Cost-Effectiveness of CDK 4/6 Inhibitors in the Treatment of HR+/HER2- Breast Cancer: A Systematic Review. Value Health.

[122] Zhu L., Wang M., Luo X., Li H., Shan H., Du Q., Zhai Q. (2022). Pharmacoeconomic Evaluations of CDK4/6 Inhibitors plus Endocrine Therapy for Advanced Hormone Receptor-Positive (HR+) and Human Epidermal Growth Factor Receptor-2 Negative (HER2−) Breast Cancer: A Systematic Review. Ann. Transl. Med..

[123] Reinert T., Pellegrini R., Barrios C.H. (2020). Lack of Access to CDK4/6 Inhibitors for Premenopausal Patients with Metastatic Breast Cancer in Brazil: Estimation of the Number of Premature Deaths. Ecancermedicalscience.

[124] Hong J., Chen T., Ouyang L., Du N., Li A., Zhou Z., Zhang H., Xia Z., Meng J. (2024). Cost-Effectiveness Comparison of Dalpiciclib and Abemaciclib Combined with an Aromatase Inhibitor as First-Line Treatment for HR+/HER2− Advanced Breast Cancer. Expert Rev. Pharmacoecon. Outcomes Res..

[125] (2025). NCCN Clinical Practice Guidelines in Oncology (NCCN Guidelines^®^) for Breast Cancer V.4.2025. https://www.nccn.org/guidelines/guidelines-detail?id=1419.

[126] Tricco A.C., Lillie E., Zarin W., O′Brien K.K., Colquhoun H., Levac D., Moher D., Peters M.D.J., Horsley T., Weeks L. (2018). PRISMA Extension for Scoping Reviews (PRISMAScR): Checklist and Explanation. Ann. Intern. Med..

